# PU.1 restores microglial dysfunction caused by C9ORF72 repeat expansions in neural organoids

**DOI:** 10.1093/brain/awaf340

**Published:** 2025-09-12

**Authors:** Tijana Ljubikj, Mayte Z Mars, Astrid T van der Geest, Channa E Jakobs, Nils Bessler, Vanessa Donega, Xynthia P R M van den Oetelaar, Marina de Wit, R Jeroen Pasterkamp

**Affiliations:** Department of Translational Neuroscience, University Medical Center Utrecht Brain Center, Utrecht University, Utrecht 3584 CG, The Netherlands; Department of Translational Neuroscience, University Medical Center Utrecht Brain Center, Utrecht University, Utrecht 3584 CG, The Netherlands; Department of Translational Neuroscience, University Medical Center Utrecht Brain Center, Utrecht University, Utrecht 3584 CG, The Netherlands; Department of Translational Neuroscience, University Medical Center Utrecht Brain Center, Utrecht University, Utrecht 3584 CG, The Netherlands; Princess Máxima Center for Pedriatic Onocology, Utrecht University, Utrecht 3584 CS, The Netherlands; Department of Translational Neuroscience, University Medical Center Utrecht Brain Center, Utrecht University, Utrecht 3584 CG, The Netherlands; Department of Anatomy and Neurosciences, Amsterdam UMC Location Vrije Universiteit Amsterdam, Amsterdam 1081 HV, The Netherlands; Amsterdam Neuroscience, Cellular and Molecular Mechanisms, Amsterdam 1081 HV, The Netherlands; Department of Translational Neuroscience, University Medical Center Utrecht Brain Center, Utrecht University, Utrecht 3584 CG, The Netherlands; Department of Translational Neuroscience, University Medical Center Utrecht Brain Center, Utrecht University, Utrecht 3584 CG, The Netherlands; Department of Translational Neuroscience, University Medical Center Utrecht Brain Center, Utrecht University, Utrecht 3584 CG, The Netherlands

**Keywords:** amyotrophic lateral sclerosis, C9ORF72, neural organoid, microglia, phagocytosis, synapse

## Abstract

Amyotrophic lateral sclerosis (ALS) is an adult-onset neurodegenerative disease characterized by loss of upper and lower motor neurons and progressive muscle wasting. Accumulating evidence indicates a role for non-neuronal cells in ALS pathogenesis, but their exact role and mechanism-of-action remain incompletely understood. A hexanucleotide (GGGGCC) repeat expansion (HRE) in *C9ORF72* is the most common genetic cause of ALS (C9-ALS) and a frequent cause of frontotemporal dementia (FTD).

Several lines of experimental evidence support a role for the immune system and microglia in C9-ALS/FTD, and depending on experimental settings and species used, both reduced and increased microglial activity have been reported. To further study microglia in C9-ALS/FTD in the context of a complex, 3D disease environment, we developed cerebral organoids that innately develop microglia derived from induced pluripotent stem cells (iPSCs) of C9-ALS/FTD patients and controls.

Here, we show reduced cellular complexity and transcriptional changes in C9 neural organoid-derived microglia (C9-oMGs), involving phagocytic, lysosomal and immune response pathways. The release of inflammatory cues from C9-ALS/FTD organoids is decreased and LAMP1 expression in C9-oMGs is reduced. Functional analysis using live imaging reveals impaired phagocytosis by C9-oMGs and reduced engulfment of the post-synaptic protein PSD-95 by C9-oMGs in organoids. Finally, our transcriptomics analysis identifies a PU.1 (encoded by *SPI1*) regulon as the most strongly downregulated transcription factor network in C9-oMGs. Viral overexpression of PU.1 rescues phagocytosis and gene expression defects in C9-microglia.

Overall, our data demonstrate reduced microglial functions in a complex cellular disease environment and identify PU.1 as a potential target for restoring microglia changes in C9-ALS/FTD.

## Teaser

Accumulating evidence implicates non-neuronal cells in ALS pathogenesis. Using patient-derived cerebral organoids, Ljubikj *et al.* reveal reduced microglial activity in C9ORF72-ALS/FTD and identify PU.1 as a key deregulated factor, restoration of which helps to rescue microglial function.

## Introduction

Amyotrophic lateral sclerosis (ALS) is an adult-onset neurodegenerative disease characterized by motor neuron (MNs) loss leading to muscle weakness and death.^1^ A hexanucleotide (GGGGCC) repeat expansion (HRE) in *C9ORF72* is the most common genetic cause of ALS (C9-ALS) and a frequent cause of frontotemporal dementia (FTD).^2,3^ C9-HREs cause ALS/FTD through different mechanisms: C9ORF72 haploinsufficiency, the formation of RNA foci and dipeptide repeat proteins (DPRs), and TDP-43 pathology leading to incompletely understood downstream defects in different cell types.^4^

Several lines of experimental evidence support a role for the immune system and microglial changes in C9-ALS/FTD.^5^ For example, human post-mortem studies describe neuroinflammation and microglial tissue infiltration.^6-9^  *C9ORF72* is strongly expressed in peripheral myeloid cells and microglia, and loss of *C9orf72* in knockout mice leads to lysosomal accumulation and altered pro-inflammatory responses in myeloid cells and microglia.^10-12^ Other work shows that mouse microglia that lack C9ORF72 transition from a homeostatic to an inflammatory state, and that C9ORF72 depletion in a mouse model of amyloid accumulation causes microglia-mediated synapse loss and neuronal deficits.^13^ Furthermore, recent single-cell transcriptomic analysis of microglia from post-mortem brain and spinal cord tissue of C9-ALS/FTD, sporadic ALS and healthy individuals reports an impaired transition of microglia towards a reactive cell state that is predicted to lead to a reduction in the microglial response to disease.^14^

Although the molecular mechanisms underlying these changes and their functional consequences remain incompletely understood, a few recent studies have used induced pluripotent stem cell (iPSC)-derived human microglia to study the effects of C9-HRE in this cell type. One study demonstrated impaired microglial phagocytosis, an exaggerated immune response upon stimulation with lipopolysaccharide (LPS) and defective initiation of autophagy as a result of *C9ORF72* haploinsufficiency.^15^ Other work identified gene expression changes in C9-ALS/FTD iPSC-derived microglia and a toxic effect of these microglia on spinal MNs upon LPS stimulation correlating with an upregulation of matrix metalloproteinase-9 (MMP-9).^16^ In contrast, another study, using iPSC-derived microglia monocultures, did not observe obvious functional and transcriptional changes in C9-ALS/FTD lines, although *C9ORF72* RNA foci, DPRs and reduced C9ORF72 levels were found.^17^ Thus, although recent studies provide important insight into how C9-HRE affect microglia, apparent discrepancies exist. For example, differences in the absence, presence or direction of specific molecular phenotypes (e.g. transcriptional changes) or functional defects [e.g. decreased phagocytosis (human) and enhanced microglia-mediated synapse loss (mouse)]. There are several possible explanations for these differences. Microglia normally function with other cell types in a 3D diseased environment. A lack of other cell types and/or the 2D environment could therefore, affect microglial function. Furthermore, mouse and human microglia differ from each other^18-20^ and microglia derived from (post-mortem) brain tissues may be more mature than those studied in culture, but also provide different insights into the disease process (i.e. disease end-stage versus developmental stage).

Therefore, we developed and used an unguided neural (cerebral) organoid model in which microglia develop innately alongside other cell types, such as neurons and astrocytes, to explore some of these differences and to further characterize the effect of C9-HRE on human microglial function. Organoid-derived microglia (oMG) show ramified morphology, closely mimic the transcriptome and functional responses of adult microglia acutely isolated from post-mortem human brain tissue, and provide the opportunity of studying microglia in the presence of other cell types (e.g. neurons and astrocytes).^21^ Our analysis of C9-ALS/FTD oMGs showed reduced microglia complexity and downregulation of genes involved in phagocytosis, lysosomes and the inflammatory response. In line with this, secretion of inflammatory cues from oMG-containing organoids and LAMP1 expression in oMGs were decreased. Further, isolated oMGs displayed a reduced ability to phagocytose bioparticles and contained less post-synaptic proteins in the organoids. RNA sequencing (RNA-seq) analysis identified a PU.1 (*SPI1*) regulon as the most strongly deregulated transcription factor (TF) network in C9-oMGs and viral-mediated expression of PU.1 in microglial cultures rescued phagocytotic and gene expression deficits. Overall, our data show that C9 microglia show reduced complexity and activity in a 3D multicellular disease environment and identify PU.1 as a potential target for activating, at least in part, these affected microglial functions.

## Materials and methods

### iPSC and cerebral organoid culture

For more details regarding the Materials and Methods see the Supplementary methods. All subjects in this study provided written informed consent and generation of iPSC lines was approved by the Ethical Medical Committee of the University Medical Center Utrecht. Control iPSC lines were derived from donors without a psychiatric or neurologic diagnosis (Supplementary Table 1). Healthy control (HC) cells had <30 HRE repeats on both alleles, while C9-ALS/FTD (C9) iPSCs had an expanded repeat on one allele with a median length between 809–1175 repeats. Neural organoids were generated using a modified version of a previously published protocol.^22^

### Single cell preparation and magnetic cell sorting

Organoid dissociation was performed as described before.^21^ Magnetic-activated cell sorting (MACS) was used to enrich for the CD11b^+^ fraction (Miltenyi, 130049601) according to the manufacturer's protocol. The CD11b^+^ cell fraction was collected in microglia culture medium [RPMI 1640 (Life Technologies, 21875034), 10% fetal bovine serum, 2 mM L-glutamine, 100 U/ml penicillin, 100 μg/ml streptomycin (BioWhittaker) and 100 ng/ml IL-34 (Pepro Tech, 200-34)]. On average, CD11b^+^ cells constituted 3%–5% of the cell suspension. Isolated CD11b^+^ cells were collected for RNA isolation or plated on poly-L-lysine-coated plates for further culture.

### LPS stimulation and multiplex cytokine detection

Whole organoids and MACS-isolated oMGs were stimulated with LPS. After 1 day in culture, oMGs were stimulated with 100 ng/ml LPS for 6 h followed by collection of media for cytokine/chemokine analysis. Cells were lysed in 500 μl Tryzol reagent for RNA isolation. For whole organoid stimulation, five organoids per line were stimulated with 100 ng/ml LPS for 24 h, to allow penetrance of LPS into organoid tissue. After 24 h, the medium was collected and CD11b^+^ cells were isolated using MACS. Isolated oMGs were lysed in 500 μl Tryzol reagent. Detected cytokine and chemokine amounts per line are available in Supplementary Table 2.

### Generation and transduction of iPSC-derived microglia

iPSCs were directly differentiated into microglia using a previously published protocol.^23^ For viral transduction, microglial progenitors were seeded at a density of 200 000 cells per well and the next day cells were infected with pLV[Exp]-EGFP (Enhanced green fluorescent protein)/Puro-EF1A>hSPI1[NM_001080547.2] (VectorBuilder, VB900006-2455hhj) or pLV[Exp]-CMV (Cytomegalovirus)>EGFP(ns):T2A:Puro (VectorBuilder; VB221214-1988tev) at MOI (multiplicity of infection) 2–5. After 24 h, the virus-containing medium was replaced with pluripotent stem cell microglia medium. Four days after infection, puromycin selection (1 μg/ml) was started for 2 days and cells were maintained in culture for up to 2 weeks.

### Phagocytosis assay and analysis

Isolated oMGs and iMGs were used in phagocytosis assays. oMGs were MACS-isolated from 10–12 Day 64 organoids per line (three healthy control and three C9 iPSC lines). Isolated oMGs or iMGs were plated in microglia medium, supplemented with 100 ng/ml IL-34 (Pepro Tech, 200-34) (100 000 cells/well). Two (oMGs) or 14 (iMGs) days after plating, pH-conjugated *Escherichia*  *coli* bioparticles (Thermo Fisher, P35361) were added (0.2 mg/ml) followed by live imaging (Leica DMi8 Thunder microscope, 10× objective) at 37°C and 5% CO_2_.

### Real-time quantitative PCR, immunohistochemistry, western blot analysis and fluorescent *in situ* hybridization

RNA extraction, cDNA synthesis and real-time quantitative PCR (RT-qPCR) were performed as described before.^22^ For primers used see Supplementary Table 3. For immunohistochemistry, 20 μm-thick sections were obtained from organoids and immunostained as described previously.^21,22,24^ For antibodies used see Supplementary Table 4. To determine C9ORF72 levels, Day 64 oMGs and 2-week matured iMGs were collected and lysed in Radioimmunoprecipitation Assay Buffer (RIPA) buffer and protein samples were analysed as described before.^22^ Locked nucleic acid (LNA) fluorescence *in situ* histochemistry (FISH) was performed on 20 μm thick organoid sections as previously described.^25^

### RNA sequencing

#### Sample preparation and RNA sequencing

Sample quality control, library preparation and sequencing were performed by GenomeScan BV [RNA Quality Number (RQN) values > 9]. Sample preparation was performed according to ‘NEBNext Ultra II Directional RNA Library Prep Kit for Illumina’ (NEB #E7760S/L).

#### Differential gene expression analysis

DESeq2 (v.1.40.2) was used for gene expression analysis in R (v.4.3.2). Counts were normalized using rlog or vst data transformation. Distance between samples was visualized using plotPCA on rlog transformed counts. Gene expression levels were corrected for batch effects. Using the Benjamini–Hochberg false discovery rate (FDR)-adjusted *P*-values with a cut-off *P* < 0.05 or *P* < 0.01 and a log_2_ fold-change (FC) > 1 and log_2_FC < 1 were considered differentially expressed genes (DEGs). Visualization of data was done using the ggplot2 library (v.3.4.4). Volcano plots visualizing the log_2_FC and –log_10_P for each gene were generated using a Bioconductor package, EnhancedVolcano (v.1.18.0).

#### Pathway enrichment analysis and regulon analysis

Pathway enrichment analysis was performed using clusterProfiler (v.4.7.1) in R using DEGs with *P* < 0.01 and *P* < 0.05. Upregulated and downregulated DEG datasets were analysed separately and over-representation analysis (ORA) using both a Gene Ontology (GO) annotation and the Kyoto Encyclopedia of Genes and Genomes (KEGG) pathway database was applied. Pathway enrichment was visualized using dotplot from the ggplot2 package and cnetplot to depict linkage between biological terms and genes. Assessment of transcription factor–target gene interaction was carried out using DoRothEA (v.1.12.0) in R. Regulons from the confidence levels A and B were assigned.

### Microglial analysis

To analyse microglia morphology, sections were stained for IBA1 (Wako Chemicals, 019–19741) and with 4′,6-diamidin-2-phenylindol (DAPI). Microglia morphology was assessed using a macro in Fiji software 1.53q and the maximum intensity projection of the IBA1 channel. To assess PSD-95 content, sections were stained for MAP2 (a dendritic marker), PSD-95 (a post-synaptic marker), IBA1 (a microglial marker) and DAPI (Supplementary Table 4). Images were taken in MAP2-rich areas where microglia were present and analysed using Imaris. To evaluate the amount of lysosomal content in oMGs, sections were (immune)stained for LAMP1, IBA1 and DAPI (Supplementary Table 4). The maximum intensity projection of the IBA1 channel was used to set a region of interest (ROI) in FIJI.

### Statistics

Statistical comparisons were performed with two-way ANOVA and Mann–Whitney tests (non-paired data) using GraphPad Prism software version 9, as normality assumptions were not met. Data are presented as single data points and means ± standard deviation. Differences were considered significant when *P* < 0.05. Immunofluorescent images were analysed and quantified by using FIJI version 1.53c software (NIH, Bethesda, MD) and Imaris software (v.9.8.0). Data were plotted using GraphPad Prism software version 9. Assembly of figures was performed using Adobe Illustrator.

## Results

### C9-ALS/FTD oMGs display morphological changes

**Figure 1:**
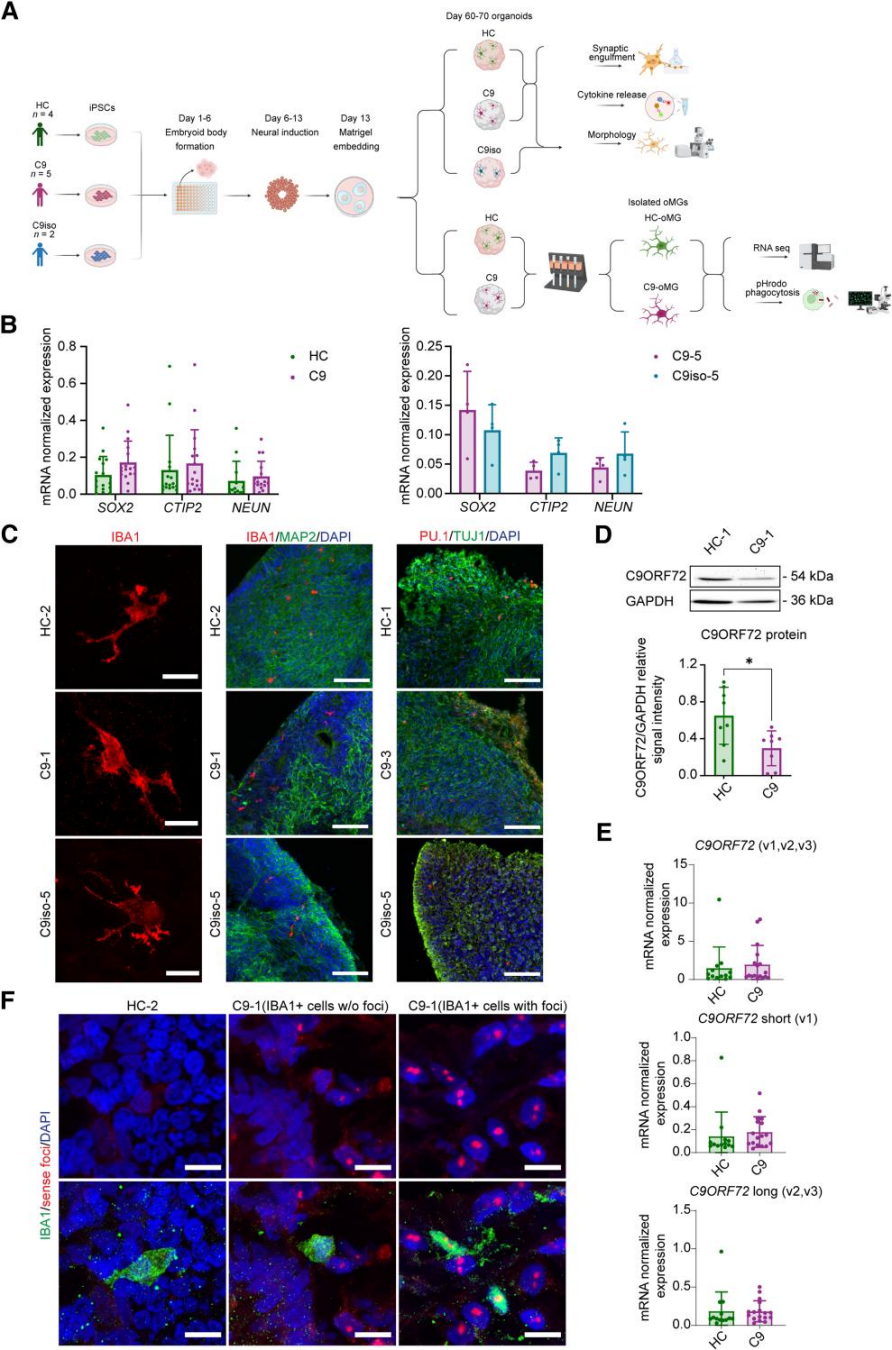
**Characterization of microglia-containing organoids from healthy and isogenic control and C9ORF72-ALS/FTD iPSCs.** (**A**) Schematic representation of the protocol used to generate cerebral organoids from the indicated iPSC lines and subsequent experimental procedures. Generated using Biorender.com. (**B)** RT-q PCR for *SOX2*, *NEUN* and *CTIP2* normalized to the housekeeping genes *RPII* and *TBP* for healthy control (HC; *n* = 4 lines, 2–3 differentiations) and C9-ALS/FTD (C9; *n* = 4 lines, 3 differentiations) (*left*), and isogenic control with parental line (C9–5 and C9iso-5, 3 differentiations) (*right*). Single data points represent each differentiation per line and means ± SD. Mann–Whitney test, ns. (**C**) Representative images showing immunohistochemistry for the microglia markers IBA1 (*left*) and IBA1 and PU.1 (*right*) in combination with MAP2 and DAPI on sections of DIV64 organoids from HC, C9 and C9iso iPSCs. Scale bars = 15 μm (*left*) and 100 µm (*right*). (**D**) *Top*: Western blot for C9ORF72 and GAPDH on DIV64 HC or C9 organoid-derived microglia (oMGs). *Bottom*: Quantification of C9ORF72 expression normalized to GAPDH as shown in *top* panel (*n* = 3 lines per condition, 3 differentiations). Single data points showing each differentiation per line and means ± SD. Mann–Whitney test, *P < 0.05. (**E**) RT-qPCR for *C9ORF72* and *C9ORF72* variants [*C9ORF72-short(v1)* and *C9ORF72-long(v2, v3*)] normalized to the housekeeping gene *RPII* for HC oMGs (HC; *n* = 3 lines, 3–6 differentiations) and C9-ALS/FTD oMGs (C9; *n* = 4 lines, 1–7 differentiations). Single data points represent each differentiation per line and means ± SD. Mann–Whitney test, ns. (**F**) Representative images showing immunohistochemistry for the microglia marker IBA1 in combination with *in situ* hybridization for RNA foci (sense) and DAPI on DIV64 C9 and HC organoids from C9 and HC. Examples of microglia with and without RNA foci are shown. Scale bars = 10 μm. Created in BioRender. Pasterkamp, J. (2025) https://BioRender.com/qv9jfpk. ALS = amyotrophic lateral sclerosis; DAPI = 4′,6-diamidino-2-phenylindole; DIV = days *in vitro*; FTD = frontotemporal dementia; GAPDH = glyceraldehyde 3-phosphate dehydrogenase; iPSC = induced pluripotent stem cell; MAP2 = microtubule-associated protein 2; ns = not significant; oMGs = organoid-derived microglia; RT-q = Real time quantitative; SD = standard deviation.

To study C9-ALS/FTD human microglia in a complex 3D environment, unguided neural (cerebral) organoids that innately develop microglia were generated.^21^ Organoids were generated from iPSC lines derived from five C9-ALS/FTD (C9) patients, four healthy control (HC) donors and two isogenic control lines (Fig. 1A and Supplementary Table 1). All iPSC lines could be differentiated into organoids and expressed the neuroepithelial marker SOX2 and neuronal markers CTIP2, NEUN and MAP2 as assessed by RT-qPCR and immunostaining at 64 days *in vitro* (DIV) (Fig. 1B and Supplementary Fig. 1A). We chose to analyse oMGs around DIV60, as microglia cells have populated organoids at this stage and show their characteristic mature ramified morphology.^21^ Furthermore, transcriptomic comparison of microglia derived from different sources (fetal, post-mortem adult, iPSC derived) showed that oMGs strongly resemble adult post-mortem microglia from DIV52 onwards.^21^ Microglia were detected in organoids generated from all iPSC lines and expressed the microglia markers IBA1 and PU.1 (Fig. 1C). oMGs were isolated by MACS and a comparable number of oMGs was observed in HC (3.43% of all cells) and C9 organoids (2.72% of all cells) (Supplementary Fig. 1B). In contrast, a significantly lower number of oMGs was purified from C9 isogenic control organoids (Supplementary Fig. 1B). This prevented us from performing some of the analyses in this study on isogenic control oMGs. Finally, the two strongest types of molecular pathology associated with C9-HRE^4^ reported in microglia were examined in oMGs.^4,26,27^ First, western blot analysis showed a ∼42% reduction in C9ORF72 protein expression in purified oMGs from C9 as compared with HC organoids (Fig. 1D and Supplementary Fig. 1C). Interestingly, no change in C9ORF72 expression was found in flowthrough lysates of the same samples, suggesting that oMGs drive the reduction in C9ORF72 levels in the organoids (Supplementary Fig. 1D). RT-qPCR assessment of C9ORF72 mRNA [overall and of specific variants (short-v1 and long-v2, v3)] did not show differences between HC-oMGs and C9-oMGs (Fig. 1E). This is in line with recent work showing reduced C9ORF72 protein but not mRNA expression in iPSC-derived microglia.^16^ Second, FISH analysis confirmed the presence of RNA foci in neurons and in 11% of microglia in C9 organoids (Fig. 1F and Supplementary Fig. 1E).

**Figure 2:**
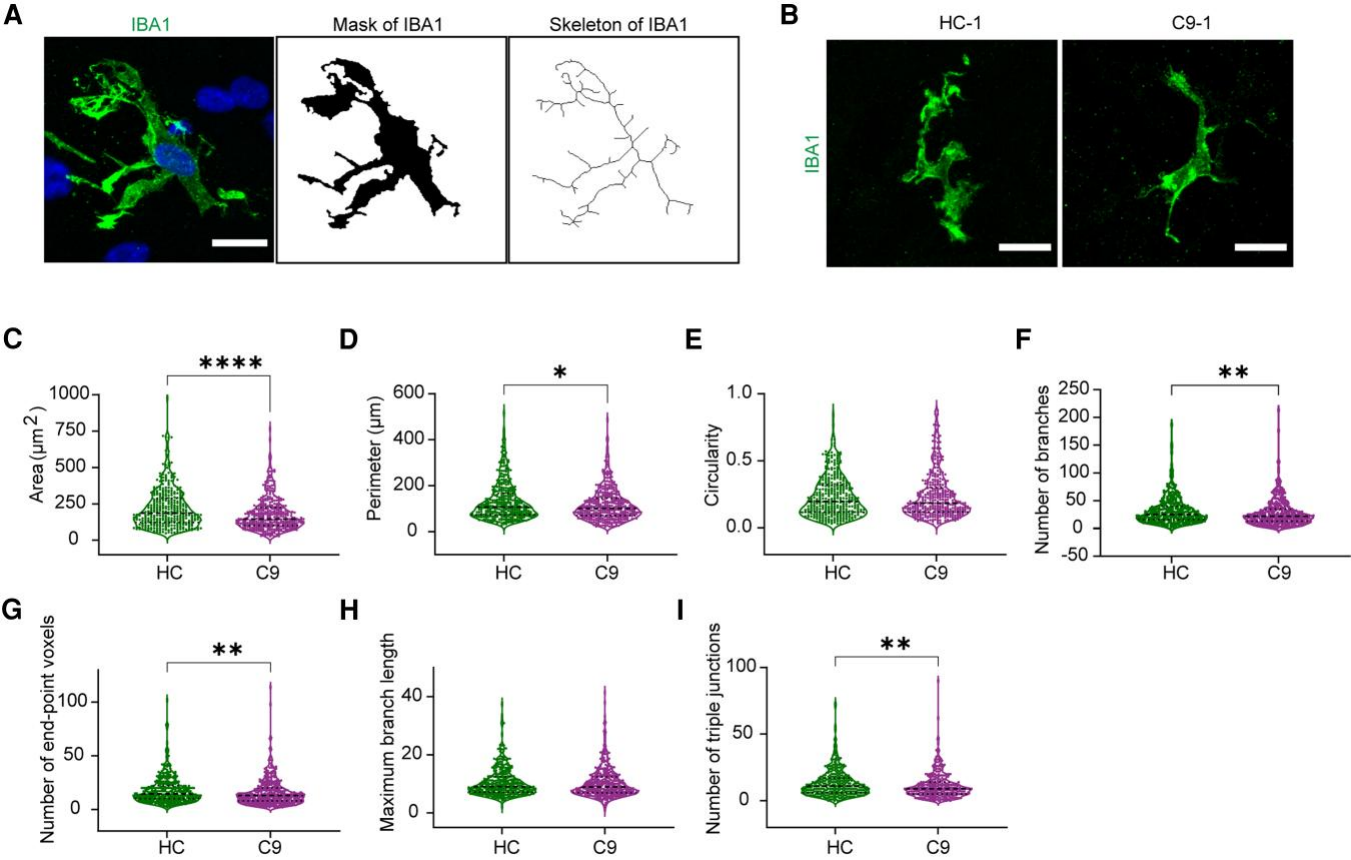
**C9ORF72-ALS/FTD organoid-derived microglia show reduced cellular complexity.** (**A**) Experimental approach to generate a skeleton of organoid-derived microglia (oMG) for the quantification of morphology. Immunohistochemistry for IBA1(*left*), mask based on IBA1 immunosignal (*middle*) and skeleton of IBA1 cell (*right*). (**B**) Immunostaining for IBA1 on DIV64 organoid sections generated from C9-ALS/FTD (C9) and healthy control (HC) oMGs. Scale bar = 15 μm. (**C**–**I**) Quantification of different morphological features from oMG skeletons from C9 (380 cells from *n* = 3 lines, 3 differentiations) and HC (363 cells from *n* = 4 lines, 3 differentiations) organoids (for area, perimeter and circularity); C9 (380 cells from *n* = 3 lines, 3 differentiations) and HC (358 cells from *n* = 4 lines, 3 differentiations) for other parameters. Data show cells and means ± SD. Mann–Whitney test, *P < 0.05, **P < 0.01 and ***P < 0.001. ALS = amyotrophic lateral sclerosis; DIV = days *in vitro*; FTD = frontotemporal dementia; SD = standard deviation.

Compared with 2D iPSC-derived microglia cultures, oMGs showed a more complex, ramified morphology (Fig. 2A). Microglia morphology is altered in several brain diseases and related to microglial function,^28^ but the effect of C9-HRE on human microglial ramification is unknown. Therefore, different morphometric parameters were measured in IBA1^+^ oMGs in DIV64 organoids (Fig. 2A and B). This revealed a small, but significant reduction in cellular area (*P* < 0.0001), perimeter (*P* = 0.0367), number of branches (*P* = 0.0021), end point voxels (*P* = 0.0013) and triple junction points (*P* = 0.0021) in C9 as compared with HC-oMGs (Fig. 2C–I). These morphological changes in C9-oMGs were confirmed when compared with isogenic control oMGs [cellular area (*P* < 0.0001), perimeter (*P* = 0.0029), number of branches (*P* = 0.0477), maximum branch length (*P* < 0.0001) and triple junction points (*P* = 0.0183)] (Supplementary Fig. 1F–L). Overall, these data show that C9-oMGs are less ramified and have smaller and more ameboid cell bodies.

### Changes in phagocytosis, lysosomal and immune response pathways in C9-ALS/FTD oMGs

**Figure 3:**
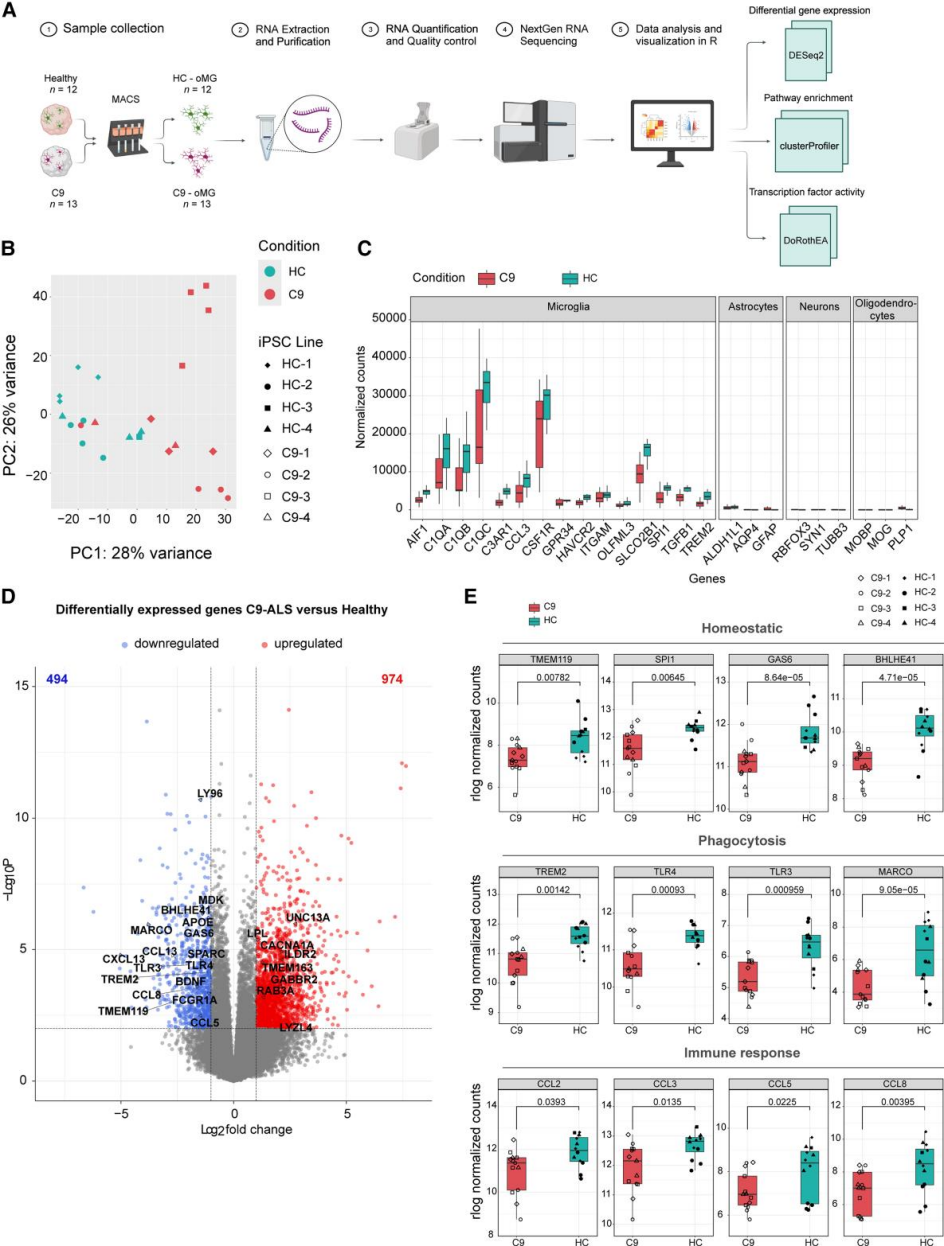
**RNA sequencing analysis of C9ORF72-ALS/FTD organoid-derived microglia reveals changes in microglia-related genes.** (**A**) Schematic representation of the bulk RNA-seq workflow and software analysis. oMGs were isolated using MACS from DIV64 cerebral organoids and total RNA was collected from healthy control (HC) lines (*n* = 4; HC-1, HC-2, HC-3, HC-4) and C9-ALS/FTD (C9) lines (*n* = 4; C9-1, C9-2, C9-3, C9-4). Microglia were acquired from 1–4 independent experiments per line (HC-1 *n* = 4 differentiations; HC-2 *n* = 4 differentiations; HC-3 *n* = 1 differentiations; HC-4 *n* = 3 differentiations; C9-1 *n* = 3 differentiations; C9-2 *n* = 4 differentiations; C9-3 *n* = 4 differentiations; C9-4 *n* = 2 differentiations). In total 25 samples (12 HC and 13 C9) were sent for RNA-seq. Generated using Biorender.com. (**B**) Principal component analysis plot of rlog normalized counts shows sample clustering based on genotype (HC, C9). (**C**) Graph showing normalized counts from the RNA-seq analysis for canonical microglia, astrocyte, neuron and oligodendrocyte genes in MACS-purified HC and C9-oMGs. (**D**) Volcano plot showing differentially expressed genes (DEGs) between C9 and HC-oMGs; 494 downregulated genes [log_2_FC < 1 and *P*_adj_ < 0.01 (in blue)] and 974 upregulated genes [log_2_FC < 1 and *P*_adj_ < 0.01 (in red)] were detected in C9-oMGs as compared with HC. A selection of DEGs is indicated. (**E**) Plots of rlog normalized counts showing changes in genes linked to homeostatic microglia (*TMEM119*, *SPI1*, *GAS6*, *BHLHE41*), phagocytosis (*TREM2*, *TLR4*, *TLR3*, *MARCO*) and immune response (*CCL2*, *CCL3*, *CCL5*, *CCL8*) in C9-oMGs as compared with HC. Created in BioRender. Pasterkamp, J. (2025) https://BioRender.com/o13a1dz. ALS = amyotrophic lateral sclerosis; DIV = days *in vitro*; FC = fold-change; FTD = frontotemporal dementia; MACS = magnetic-activated cell sorting; oMGs = organoid-derived microglia; RNA-seq = RNA sequencing; SD = standard deviation.

Altered microglia morphology may indicate changes in the activation state of these cells.^28-30^ To evaluate whether such changes were also reflected at the molecular level, RNA sequencing was performed on oMGs from HC (HC-1-4) and C9 organoids (C9-1-4) (Fig. 3A). Principal component analysis (PCA) based on gene expression revealed segregation of samples based on disease background (C9 versus HC) (Fig. 3B). To confirm the purity of the samples and exclude C9-ALS/FTD-related effects on microglial differentiation, normalized counts for known markers of different cell types^31-33^ were plotted. Various canonical microglia markers^31,34-36^ were enriched in HC- and C9-oMG samples, whereas genes normally expressed in astrocytes (*ALDH1L1, AQP4, GFAP*), neurons (*REBFOX3, SYN1, TUBB3*) and oligodendrocytes (*MOBP, MOG, PLP1*) were largely absent (Fig. 3C).

Next, differential gene expression analysis was performed. This analysis revealed 1468 DEGs in C9- as compared with HC-oMGs (*P*_adj_ < 0.01, log_2_FC > ± 1; 974 upregulated and 494 downregulated genes) (Fig. 3D and Supplementary Table 5). Interestingly, important microglia genes were downregulated in C9-oMGs, including genes related to microglia homeostasis^31,35^ (*TMEM119*, *SPI1*, *GAS6*, *BHLHE41*), phagocytosis^37^ (*TREM2*, *TLR4*, *TLR3*, *MARCO*, *SYK*, *FCGR1A*, *CD33*, *CD36*), immune response^31,38^ (*CCL2*, *CCL3*, *CCL5*, *CCL8*, *IL1R1*, *LY96*, *CCL13*, *CCL23*), lysosomes^39,40^ (*LAMP1*, *CTSB*, *CTSW*, *CTSD*) and MH-II class/antigen presentation^31,38^ (*HLA-DRB5*, *HLA-DMA*, *HLA-DQA1*, *HLA-DRA*) (Fig. 3E and Supplementary Fig. 2A). To further study microglial gene expression in the RNA-seq data, expression of genes related to homeostatic microglia and disease-associated microglia (DAM) signatures was assessed. First, expression of 70 genes described by several studies as being part of a homeostatic microglial signature were examined.^35,41,42^ Of these genes, 29 genes were differentially expressed in the RNA-seq data (*P*_adj_ < 0.05) (6 upregulated and 23 downregulated genes) (Supplementary Table 6). Second, expression of 295 genes linked to a DAM signature [including genes described as human DAM (Mic 1 subcluster or mouse DAM^43^)] were assessed. This identified 117 DEGs, most of which represented downregulated genes in C9-OMGs. Only 14 genes showed increased expression in C9-ALS oMGs (including *LYZL4, LPL, ILDR2, ADGRG1*) (Supplementary Table 7). Overall, these data show that C9-oMGs display downregulation of homeostatic genes, as well as genes implicated in phagocytosis, lysosomes, immune response and antigen presentation. Additionally, C9-oMGs exhibit an impaired DAM transition, as compared with typical DAM responses in other diseases.

**Figure 4:**
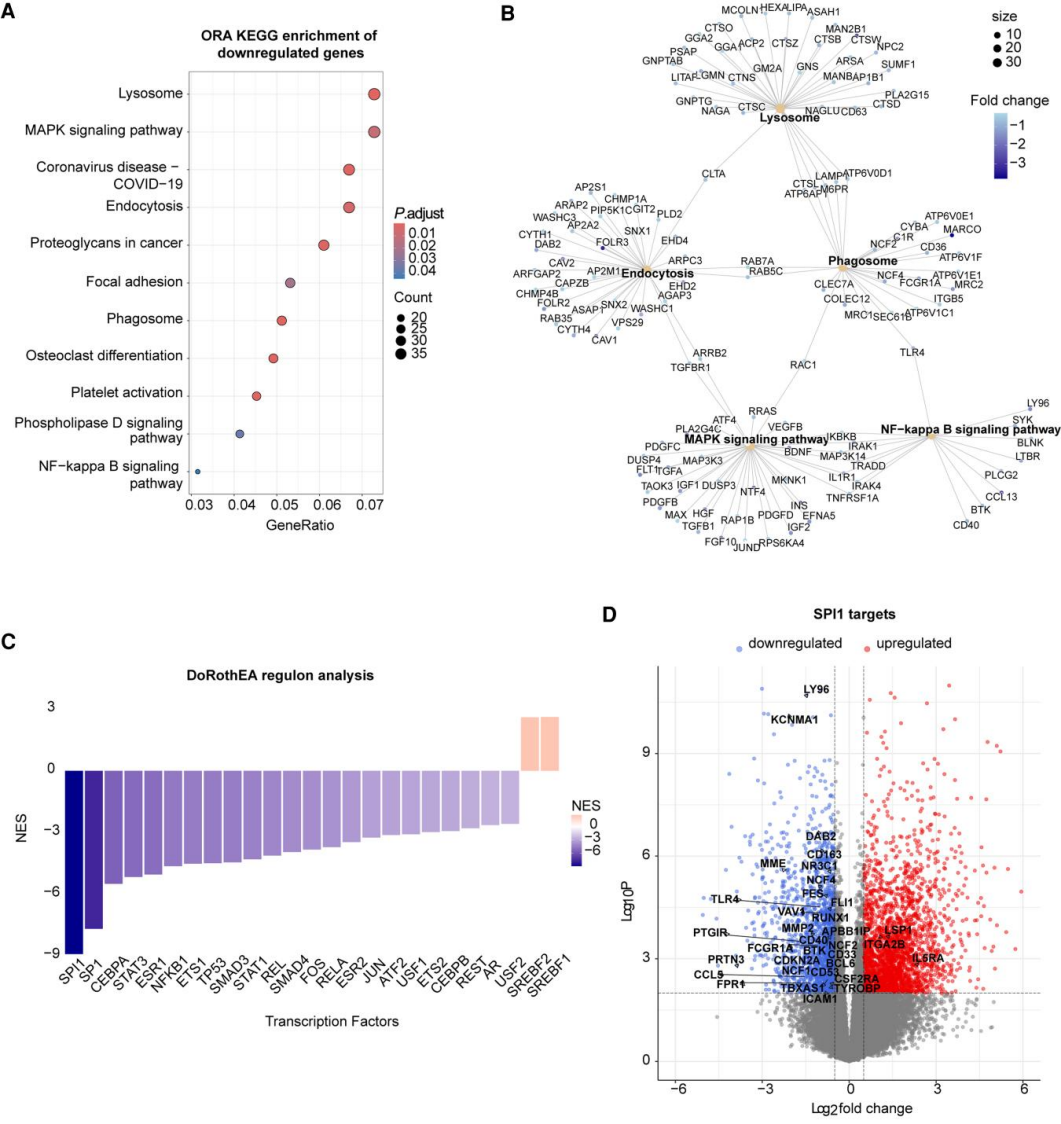
**Downregulation of phagocytic, lysosomal and immune response pathways in C9ORF72-ALS/FTD organoid-derived microglia.** (**A**) Dot plot showing the top significantly enriched KEGG pathways after over-representation analysis (ORA) of downregulated genes in C9ORF72-ALS/FTD (C9) versus healthy control (HC) organoid-derived microglia (oMGs) using the clusterProfiler package. (**B**) Net plot showing the linkage of genes with terms as a network. Dot size corresponds with *P*-value. Log_2_fold-change (FC) values for each gene are represented as colours. (**C**) Bar chart showing the most highly enriched transcription factors by their normalized enrichment score (NES) as determined by DoRothEA analysis. (**D**) Volcano plot showing changes in *SPI1* (encoding PU.1) targets identified with the DoRothEA pipeline using the RNA sequencing data (log_2_FC > 0.5 and *P*_adj_ < 0.05).ALS = amyotrophic lateral sclerosis; FTD = frontotemporal dementia; KEGG = Kyoto Encyclopedia of Genes and Genomes.

To understand more generally which biological processes and pathways were deregulated in C9-oMGs, KEGG pathway (Supplementary Table 8) and GO analysis (Supplementary Table 9) were performed on the downregulated DEGs. This identified negative enrichment for pathways and cellular components important in microglia including lysosomes, phagocytosis and inflammatory responses (Fig. 4A and B and Supplementary Fig. 3A–C). GO analysis of the upregulated DEGs revealed pathways involved in synapse assembly, regulation of synapse transmission and axon development (Supplementary Fig. 3D). However, as many of the downregulated DEGs represented microglia-related genes, we focused on these genes and associated pathways for further analysis. To assess whether changes in transcription factor (TF) activity may underlie some of the gene expression changes in C9-oMGs, DoRotheEA analysis was performed. DoRothEA is a gene regulatory network analysis pipeline that contains TF–target interactions from different types of evidence and uses statistical enrichment analysis to infer TF activities.^44^ In line with the KEGG and GO analyses (Fig. 4A and B and Supplementary Fig. 3A–C), DoRothEA analysis highlighted several TFs important for microglia development and function, including *SPI1, SP1, CEBPA, STAT3, NFKB1, SMAD3* and *JUN*^34,45^ (Fig. 4C). The PU.1 (encoded by *SPI1*) regulon was the most strongly deregulated TF network in our dataset and analysis of PU.1 targets in the RNA-seq data (*P*_adj_ < 0.05, log_2_FC > 0.5) showed downregulation of genes such as *RUNX1*, *CD33*, *TLR4*, *LY96* and *BCL6* (Fig. 4D and Supplementary Table 10). Moderate downregulation of *SPI1* was observed in C9-oMGs (Supplementary Fig. 3E). Together, our results reveal a negative enrichment for pathways involved in basic microglial functions and cellular compartments in oMGs and highlight altered TF networks that may underlie these changes.

### Reduced cytokine and chemokine release in microglia-containing C9-ALS/FTD organoids

**Figure 5:**
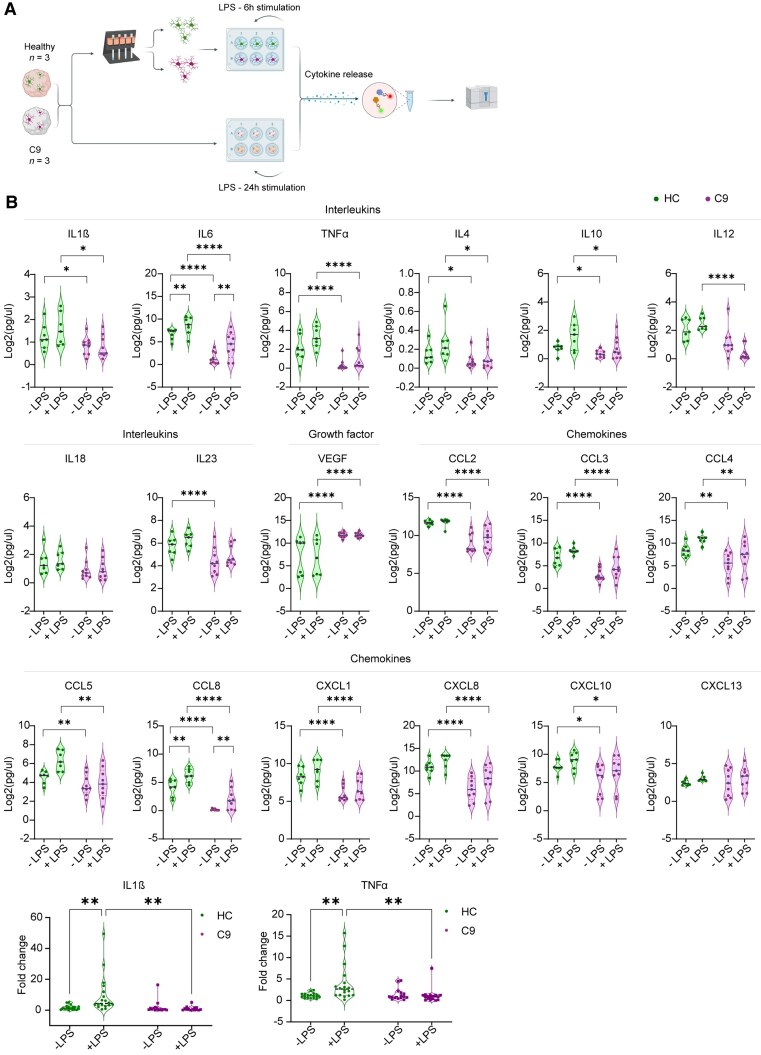
**C9ORF72-ALS/FTD microglia-containing organoids display changes in the release of cytokines and chemokines.** (**A**) Schematic representation of the experimental set-up for LPS stimulation of whole organoids and plated organoid-derived microglia (oMGs) followed by quantification of interleukins, chemokines and growth factors in the medium. Generated with Biorender.com. (**B**) Cytokine/chemokine profile analysis of media from whole organoids 24 h after stimulation with control or 100 ng/ml LPS [healthy controls (HC; *n* = 3 lines, 2–3 differentiations) and C9ORF72-ALS/FTD (C9; *n* = 3 lines, 3 differentiations)] using the Luminex xMAP Multiplex Assay technology. Data points indicate cytokine/chemokine concentrations as log_2_ (pg/μl) of each line differentiation and means ± SD. A two-way ANOVA was performed to analyse the effect of LPS stimulation and C9 disease background on cytokine/chemokine concentration, followed by Tukey's multiple comparisons test, *P < 0.05, **P < 0.01 and ***P < 0.001. (**C**) Real-time quantitative PCR analysis of oMGs isolated from organoids after whole organoid LPS stimulation for two key cytokines *(IL-1β* and *TNF-α*) normalized to the housekeeping gene *RPII* for HC (*n* = 3 lines, 2–3 differentiations) and C9-oMGs (*n* = 3 lines, 3 differentiations). Single data points show individual line differentiation and means ± SD. Two-way ANOVA followed by Tukey's multiple comparisons test, *P and < 0.05 **P < 0.01. Created in BioRender. Pasterkamp, J. (2025) https://BioRender.com/0o0ldmq.ALS = amyotrophic lateral sclerosis; FTD = frontotemporal dementia; LPS = lipopolysaccharide; SD = standard deviation.

Microglia can release pro-inflammatory (e.g. IL-1β, IL-6 and TNF-α) or anti-inflammatory cytokines (e.g. IL-4 and IL-10) in response to external stimuli and thereby contribute to neurodegenerative processes.^46,47^ The RNA-seq data showed differential expression of several cytokines and chemokines in C9-oMGs and changes in inflammatory pathways (Fig. 3E and Supplementary Fig. 3A–C). To further study this differential inflammatory response, the production of different cytokines, chemokines and growth factors was examined in HC- and C9-oMGs, in the presence or absence of the inflammatory stimulant LPS using Luminex xMAP Multiplex Assay technology. Both isolated oMGs and whole organoids were treated with LPS followed by analysis of oMG or organoid medium (Fig. 5A). Eighteen factors were analysed, which were either altered in the RNA-seq data or previously studied in the context of disease (Supplementary Table 2). In general, C9 organoids displayed lower levels of the selected pro- and anti-inflammatory cytokines and chemokines in comparison to HC organoids, both under basal conditions and after 24 h of LPS stimulation. IL-18 and chemokine (C-X-C motif) ligand (CXCL)13 were unchanged and Vascular endothelial growth factor (VEGF), a growth factor, was increased in the medium of C9 organoids (Fig. 5B). LPS stimulation did not change the production of most of the selected candidates in HC and C9 conditions, except for IL-6 and CCL8, which were increased upon LPS treatment in HC and C9 organoid medium (Fig. 5B). To assess whether some of these whole organoid effects could derive from oMGs in these organoids, RT-qPCR was performed for two candidates, *TNF-α* and *IL-1β*, two well-known pro-inflammatory cytokines, in oMGs freshly isolated from organoids following control or LPS treatment. *TNF-α* and *IL-1β* mRNA levels were increased in oMGs following LPS treatment of HC, but not C9 organoids (Fig. 5C). As the RT-qPCR analysis revealed changes in cytokine gene expression in isolated oMGs, we next assessed expression of the 18 factors in the medium of MACS-purified oMGs. In basal conditions, HC and C9-oMGs showed comparable levels of the candidates in the medium, except for CCL2 and CXCL1, which were decreased in C9 medium (Supplementary Fig. 4A). LPS stimulation caused a similar significant increase in the levels of most candidates in both HC and C9-oMGs, but no response was observed for CCL2, CXCL8 and VEGF in HC or C9 conditions (Supplementary Fig. 4A). In line with these observations, analysis of *TNF-α* and *IL-6* expression in plated oMGs, before and after LPS stimulation, showed an effect of LPS treatment, but no differences between HC and C9 cultures (Supplementary Fig. 4B).

Overall, these results show a reduction in the release of cytokines and chemokines in C9 as compared with HC organoids under basal conditions, in line with reduced expression of several of these cues in the RNA-seq data. Purified oMG cultures did not reveal changes in the basal levels of different cytokines and chemokines, but displayed robust and comparable responses to LPS in both HC and C9 cultures. LPS responses were more limited following treatment of whole organoids.

### C9-ALS/FTD oMGs show reduced uptake of synaptic proteins

In addition to changes in the immune response, our RNA-seq analysis showed downregulation of several genes related to phagocytosis (Fig. 3D and E), which is in line with the observation that iPSC-derived microglia in 2D cultures show impaired phagocytosis of pH-sensitive zymosan bioparticles.^15^ Therefore, different experimental paradigms were used to assess the phagocytotic ability of oMGs.

**Figure 6:**
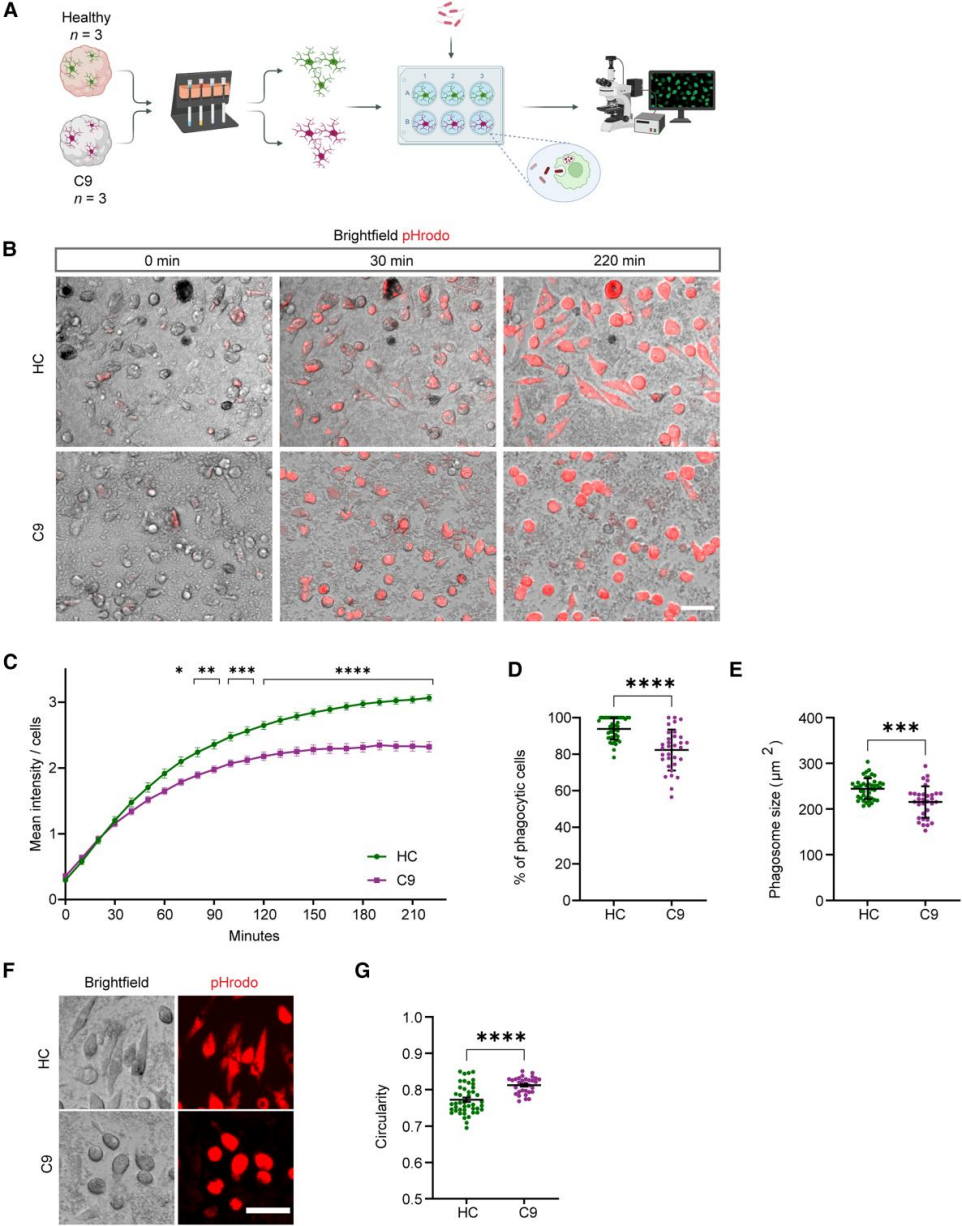
**C9ORF72-ALS/FTD organoid-derived microglia show reduced phagocytotic capacity.** (**A**) Schematic overview of the experimental set-up for analysis of the phagocytic capacity of isolated healthy control (HC) and C9ORF72-ALS/FTD organoid-derived microglia (oMG) using pH-sensitive *E.*  *coli* bioparticles (pHrodo). Generated with Biorender.com. (**B**) Representative live cell images of isolated HC and C9-oMGs at 0, 30 and 220 min incubation with pH-sensitive *E. coli* bioparticles (pHrodo, red). Scale bar = 15 μm. (**C)** Quantification of live cell imaging of pHrodo uptake, measured as mean intensity per number of cells per frame in 10 min intervals for a total of 220 min for HC (*n* = 3 lines, *n* = 2–3 differentiations, *n* = 4 image frames per line, per differentiation) and C9 (*n* = 3 lines, *n* = 2 differentiations, *n* = 4 image frames per line, per differentiation). Data are represented as mean ± SD; Two-way ANOVA and Šidák multiple comparisons test, *P < 0.05, **P < 0.01 and ***P < 0.001. (**D**) Quantification of the percentage of phagocytic active cells represented as the number of fluorescent cells normalized to the number of total cells at 220 min of scanning, for HC (*n* = 3 lines, *n* = 2–3 differentiations) and C9 (*n* = 3 lines, *n* = 2 differentiations). (**E**) Quantification of pHrodo area (μm^2^) normalized to the number of cells measured at 220 min of live imaging in HC (*n* = 3 lines, *n* = 2–3 differentiations) and C9-ALS (*n* = 3 lines, *n* = 2 differentiations). (**F**) Images showing cellular morphology after uptake of pHrodo particles in HC and C9-oMGs. Scale bar = 15 μm. (**G**) Quantification of cell morphology (images as in **F**) for HC (*n* = 3 lines, *n* = 2–3 differentiations) and C9 (*n* = 3 lines, *n* = 2 differentiations). (**D**–**G**) Single data points show cells and means ± SD; Mann–Whitney test, *P < 0.05, **P < 0.01 and ***P < 0.001.ALS = amyotrophic lateral sclerosis; FTD = frontotemporal dementia; SD = standard deviation.

As oMGs were reported to have a more ‘mature’ gene expression profile as compared with 2D iPSC-generated microglia,^21^ we first assessed the phagocytic capacity of HC and C9-oMGs with a 2D phagocytosis approach that was used previously.^15-17^ Purified oMGs were exposed to pH-sensitive *E. coli*-pHrodo bioparticles (pHrodo) followed by live imaging for 220 min (Fig. 6A). This analysis revealed reduced internalization of pHrodo particles by C9- as compared with HC-oMGs from 60 min onwards, and this difference increased with time (Fig. 6B and C). At 220 min, 100% of HC-oMGs had taken up pHrodo particles, whereas only 80% of C9-oMGs were pHrodo^+^ (Fig. 6D; *P*_adj_ < 0.0001). In addition, pHrodo^+^ area per number of cells was decreased in C9-oMGs (Fig. 6E; *P*_adj_ = 0.0001) and more detailed analysis revealed that C9-oMGs displayed a more rounded morphology compared with HC (Fig. 6F and G; *P*_adj_ < 0.0001). Overall, these data indicate that C9-HRE leads to a reduction in the phagocytic capacity of oMGs in 2D cultures.

**Figure 7:**
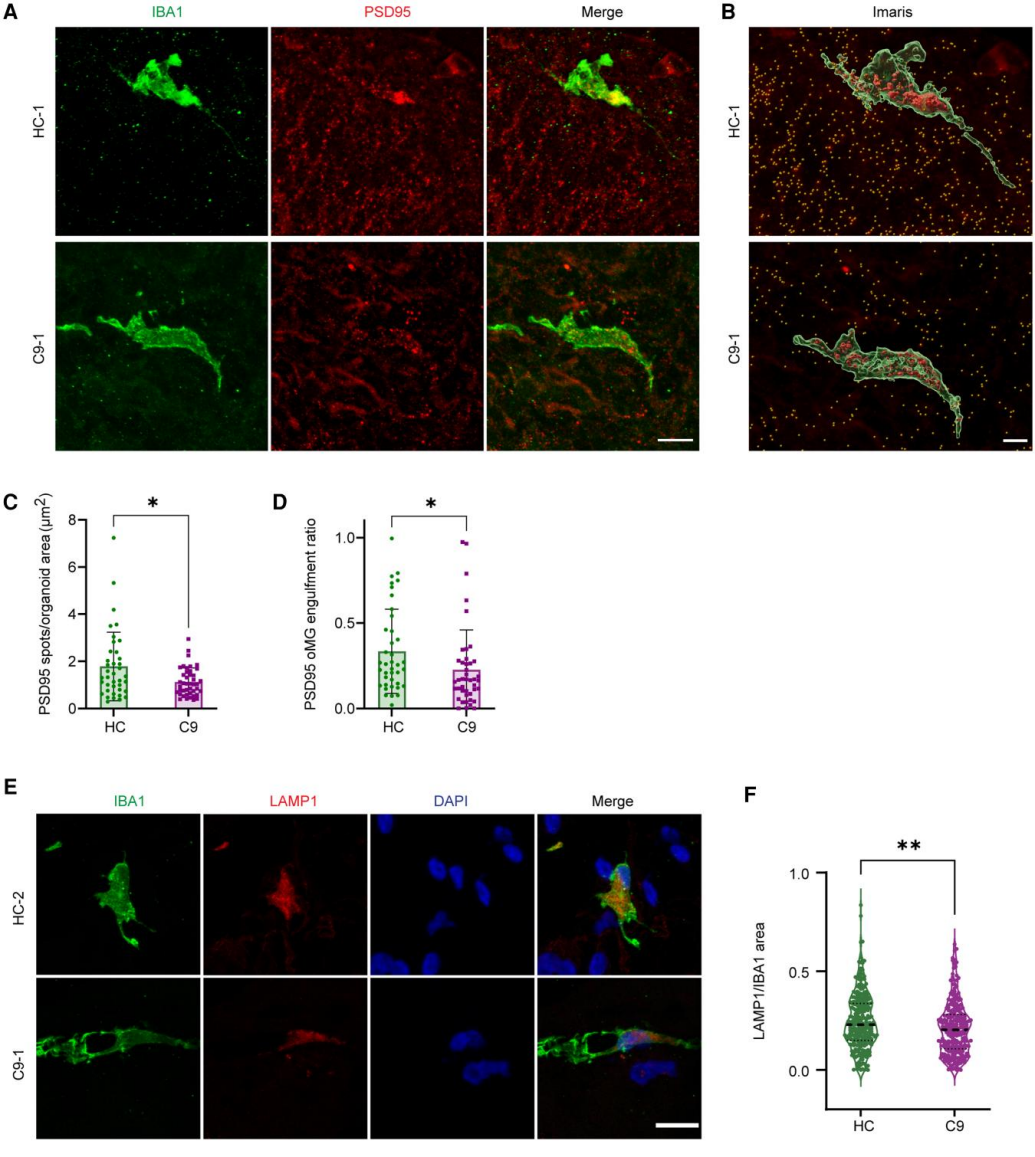
**C9ORF72-ALS/FTD organoid-derived microglia show reduced synaptic protein content and reduced LAMP1 expression in organoids.** (**A**) Representative images of immunostaining for PSD-95 (red) and IBA1 (green) in cryosections of DIV64 organoids. C9 = C9ORF72-ALS/FTD; HC = healthy control. Scale bar = 10 μm. (**B**) Imaris analysis of PSD-95 expression in organoid-derived microglia (oMG) (images as in **A**). PSD-95 internalized by IBA1-positive oMGs was quantified as a volume (red), while PSD-95 outside of microglia was marked as spots (yellow). Scale bar = 5 μm. (**C**) Quantification of the amount of PSD-95 in organoids represented as the number of PSD-95 spots outside the IBA1-positive area normalized to the image area (μm^2^) for HC organoids (*n* = 2 lines, *n* = 2–3 differentiations, *n* = 38 images in total) and C9 organoids (*n* = 3 lines, *n* = 2 differentiations, *n* = 44 images in total) at DIV64. Single data points show analysed images and means ± SD; Mann–Whitney test, *P < 0.05. (**D**) Quantification of the PSD-95 engulfment ratio as PSD-95 volume within the IBA1-positive masked area normalized to the number of PSD-95 spots outside of IBA1-positive for HC-oMGs (*n* = 2 lines, *n* = 2–3 differentiations, *n* = 39 cells in total) and C9-oMGs (*n* = 3 lines, *n* = 2 differentiations, *n* = 43 cells in total). Single data points show cells and means ± SD; Mann–Whitney test, *P < 0.05. (**E**) Representative images of immunostaining for LAMP1 (red) in IBA1-positive microglia (green) in sections of HC and C9 organoids. Scale bar = 15 μm. (**F**) Quantification of lysosome size in oMGs calculated as LAMP1-positive area per IBA1-positive cell for HC-oMGs (*n* = 3 lines, *n* = 1–3 differentiations, *n* = 280 analysed cells in total) and C9-oMGs (*n* = 4 lines, *n* = 1–3 differentiations, *n* = 254 analysed cells in total). Mann–Whitney test, single data points and means ± SD, **P < 0.01.ALS = amyotrophic lateral sclerosis; DIV = days *in vitro*; FTD = frontotemporal dementia; SD = standard deviation.

Microglia are responsible for normal maintenance of CNS tissue as well as the local response to injury or infection. Further, accumulating evidence indicates that microglia play an important role in the development and plasticity of synaptic contacts.^48^ For example, microglia can remove excess immature synapses, thereby prompting synapse and circuit maturation.^48-50^ Microglial dysfunction and subsequent effects on synaptic connectivity are being increasingly implicated in the pathogenesis of different neurodegenerative diseases. For example, mouse microglia lacking C9ORF72 can promote enhanced synapse loss and associated neuronal deficit in a mouse model of amyloid accumulation.^13^ In apparent contrast to these observations, our live imaging data (Fig. 6), and that of others,^15^ suggest that human C9 microglia have a reduced ability to phagocytose. To study this phenotype further in the context of 3D human tissue, we exploited unguided neural organoids as a 3D brain environment in which microglia develop innately with other cell types to study the ability of oMGs to take up an abundant post-synaptic protein, PSD-95 (post-synaptic density 95). Our previous work had shown that oMGs can phagocytose PSD-95 in organoids.^21^ Sections from DIV64 organoid lines were immunostained for the microglial marker IBA1, PSD-95 and the dendritic marker MAP2 (not shown) and quantified (Fig. 7A and B). To ensure microglia were in immediate contact with neurons, we only selected microglia cells in MAP2^+^ areas. The overall number of PSD-95^+^ spots was decreased in C9 as compared with HC organoids, which may reflect changes in synaptogenesis and/or loss of synapses (Fig. 7B and C).^22^ Therefore, microglial engulfment of PSD-95 was quantified as the volume of ingested PSD-95 within the IBA1 area normalized to PSD-95 puncta in the immediate environment of the microglia to correct for reduced PSD-95 puncta. This analysis revealed a decrease in PSD-95 signals in C9-oMGs in organoids (Fig. 7D).

In addition to altered phagocytosis pathways, changes in lysosomal pathways were identified in the RNA-seq analysis of oMGs. Therefore, in addition to PSD-95, we assessed LAMP1 expression in oMGs in organoids. LAMP1 is a lysosomal protein important for protein degradation, for example following phagocytosis, and is frequently affected in neurodegenerative disorders.^51^ Loss of C9ORF72 function had previously been associated with lysosomal changes, such as enlarged lysosomes, in microglia and myeloid cells.^11^ To further investigate lysosomal changes in oMGs in organoids, organoids were immunostained for IBA1 and LAMP1 (Fig. 7E). Comparison of LAMP1 expression in HC- and C9-ALS oMGs revealed a small, significant decrease in the expression of LAMP1 in C9-oMGs, which is in line with the RNA-seq data (Fig. 7F). Together, these data unveil a reduced ability of C9-oMGs for phagocytosis in 2D and 3D models, and hint at lysosomal defects.

### PU.1 overexpression rescues phagocytosis defects in C9 microglia

**Figure 8:**
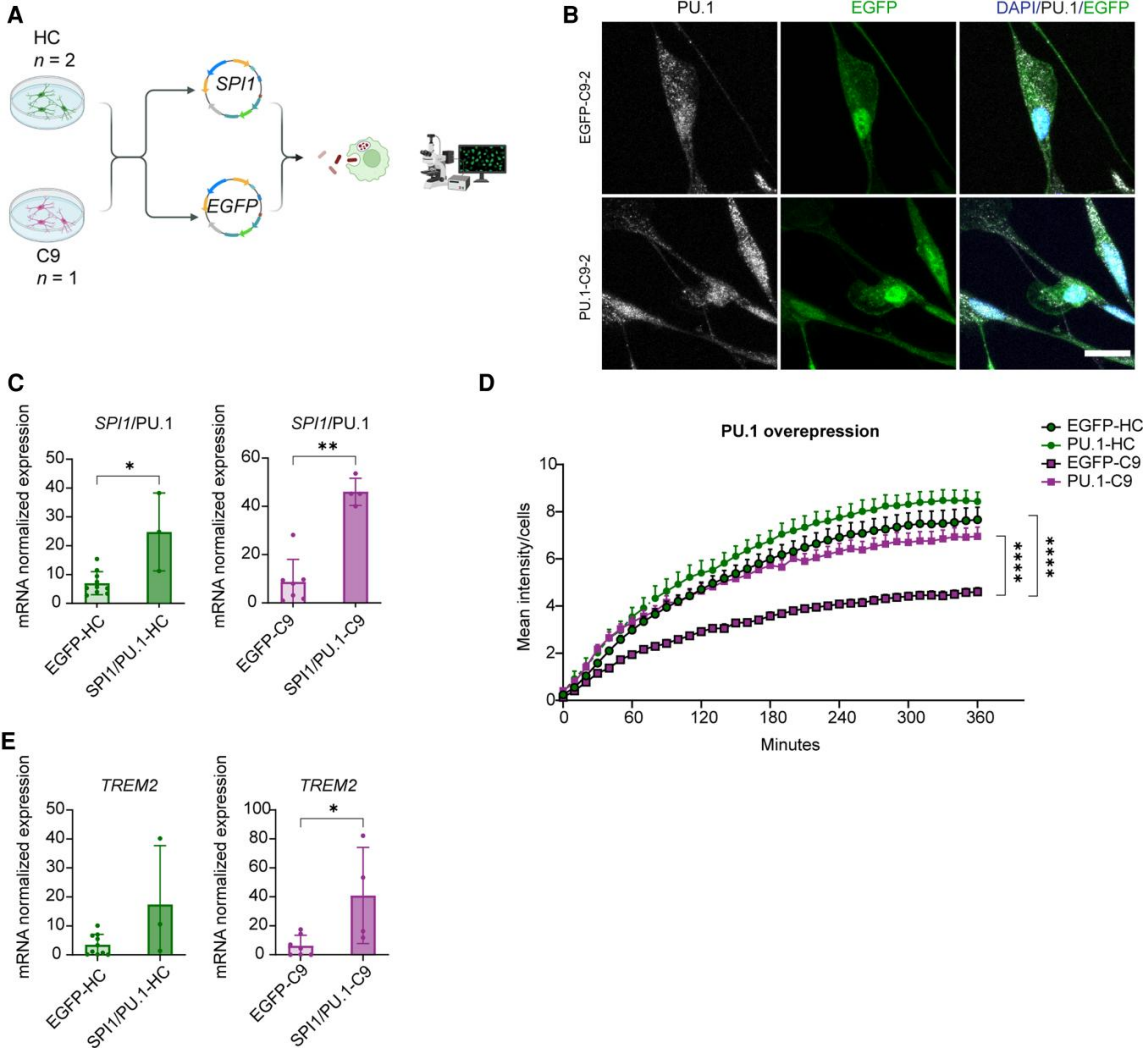
**Expression of PU.1 in C9ORF72-ALS/FTD microglia rescues phagocytic defects and *TREM2* expression.** (**A**) Schematic overview of the experimental set-up used to study the effect of PU.1 (*SPI1*) lentiviral overexpression. Generated with Biorender.com. (**B**) Representative immunofluorescent images showing expression of PU.1 protein and EGFP in C9ORF2-ALS/FTD (C9) iMGs after PU.1(*SPI1*) lentiviral transduction. Lentiviral transduction of PU.1(*SPI1*) leads to increased PU.1 expression. DAPI was used to stain all cells. Scale bar = 15 μm. (**C**) Real-time quantitative PCR (RT-qPCR) for *SPI1* normalized to the housekeeping gene *RPII* after overexpression in healthy control (HC) samples [SPI1-HC (*n* = 2 lines, 1–3 differentiations) and EGFP-HC (*n* = 2 lines, 1–2 differentiations)] and C9 samples [SPI1-C9 (*n* = 1 line, 2 differentiations) and EGFP-C9 (*n* = 1 line, 3–4 differentiations)]. Single data points showing individual lines and experimental runs and means ± SD. Lentiviral transduction of PU.1(*SPI1*) leads to increased *SPI1* expression. Mann–Whitney test, *P < 0.05, **P < 0.01. (**D**) Quantification of live cell imaging of pHrodo uptake, measured as mean intensity per number of cells per frame in 10 min intervals for a total of 360 min for SPI1-HC (*n* = 2 lines, *n* = 3 differentiations, *n* = 5 image frames per line, per differentiation), EGFP-HC (*n* = 2 lines, *n* = 3 differentiations, *n* = 4–5 image frames per line, per differentiation), SPI1-C9 (*n* = 1 line, *n* = 3 differentiations, *n* = 5 image frames per line, per differentiation) and EGFP-C9 (*n* = 1 line, *n* = 3 differentiations, *n* = 5 image frames per line, per differentiation). Data are represented as means ± standard error of the mean; two-way ANOVA and Tukey multiple comparisons test, ****P < 0.0001. (**E**) RT-qPCR for *TREM2,* a downstream target of *SPI1*, normalized to the housekeeping gene *RPII* in HC lines [SPI1-HC (*n* = 3 lines, 1–3 differentiations) and EGFP-HC (*n* = 2 lines, 1–2 differentiations)] and C9 lines [SPI1-C9 (*n* = 2 lines, 2 differentiations) and EGFP-C9 (*n* = 2 lines, 3–4 differentiations)]. Single data points showing individual lines and experimental runs and means ± SD. Mann–Whitney test, *P < 0.05. Created in BioRender. Pasterkamp, J. (2025) https://BioRender.com/rzb5ixg.ALS = amyotrophic lateral sclerosis; DAPI = 4′,6-diamidino-2-phenylindole; EGFP = enhanced green fluorescent protein; FTD = frontotemporal dementia; iMGsi = iPSC-derived microglia; PSC = induced pluripotent stem cell; SD = standard deviation.

Several of our experiments indicated that C9-oMGs have a reduced capacity for phagocytosis, in line with work performed with iPSC-derived microglia (iMGs).^15^ Interestingly, DoRothEA analysis of the RNA-seq data from C9-oMGs identified the PU.1 (*SPI1*) regulon as the most strongly deregulated TF network. Multiple PU.1 targets with established roles in phagocytosis were downregulated in C9-oMGs, including *TLR4*, *CD33*, *TREM2* (Fig. 4D). PU.1 (*SPI1*) is a key transcription factor involved in myeloid cell development and microglial gene expression.^45,52-54^ Reduced PU.1 (*SPI1*) expression causes decreased phagocytosis,^55-57^ whereas enhanced levels resulted in increased phagocytosis of zymosan particles by microglia.^55^ Based on these observations, we hypothesized that increased expression of PU.1 (*SPI1*) may be able to reverse the reduced phagocytic capacity of C9 microglia. As only relatively limited numbers of oMGs can be purified from organoids, which can be cultured for a short duration, we performed lentiviral overexpression of PU.1 (*SPI1*) in 2D iMGs cultures (Fig. 8A). C9-iMGs show mild gene expression changes, but similar to oMGs, display reduced phagocytic capacity.^15,16^ iMGs were generated using a previously published protocol (Supplementary Fig. 5A)^23^ and immunohistochemistry and RT-qPCR showed comparable expression of selected microglial markers in HC and C9-iMGs (Supplementary Fig. 5B and C). Additionally, significantly reduced expression of C9ORF72 protein was observed in C9-iMGs as compared with HC (Supplementary Fig. 5E). No change in overall C9ORF72 mRNA expression was detected, although levels of the C9-short variant (v1) were reduced in C9-iMGs in comparison to HC (Supplementary Fig. 6A) but not isogenic controls (Supplementary Fig. 6B). *SPI1* levels showed a non-significant reduction in C9-iMG samples in comparison to HC but not isogenic controls (Supplementary Fig. 5D). To examine the effect of PU.1 (*SPI1*) on phagocytosis, C9- and HC-iMGs were transduced with lentiviral vectors containing *SPI1* or *EGFP* expression cassettes (Fig. 8A). Immunohistochemistry and RT-qPCR showed elevated PU.1 (*SPI1*) expression in C9- and HC-iMGs following transduction with lentivirus expressing PU.1 (*SPI1*) (Fig. 8B and C). To examine the effect of increased PU.1 expression, transduced iMGs were analysed in pHrodo assays for 360 min. In line with our observations for C9-oMGs, EGFP-transduced C9-iMGs displayed a reduced capacity to phagocytose pHrodo particles as compared with EGFP HC-iMGs (Fig. 8D, Supplementary Fig. 6C and D). Interestingly, lentiviral PU.1 (*SPI1*) overexpression resulted in increased phagocytic activity of C9-iMGs towards HC control levels (Fig. 8D). Enhanced PU.1 (*SPI1*) expression not only affected phagocytosis but also increased gene expression downstream of PU.1 (*SPI1*), as exemplified by enhanced levels of *TREM2*, a gene downregulated in the C9-oMG RNA-seq data (Figs. 3E and 8E). However, while *TREM2* expression was elevated in both HC- and C9-iMGs, the phagocytosis-promoting effect of lentiviral PU.1 (*SPI1*) was especially robust in C9 as compared with HC iMGs (Fig. 8D). Together, these data show that increased PU.1 (*SPI1*) expression is able to reverse phagocytotic defects observed in C9 microglia.

## Discussion

In this study, we used a cerebral organoid model to further define the effects of C9-HRE on human microglial function in a complex, diseased microenvironment. Analysis of C9-ALS/FTD oMGs showed reduced morphological complexity and downregulation of genes involved in phagocytosis, lysosomes and the inflammatory response. In line with this, secretion of inflammatory cues from oMG-containing organoids was decreased and LAMP1 expression in C9-oMGs reduced. In addition, phagocytosis of bioparticles by C9-oMGs was impaired and less post-synaptic protein was detected in oMGs in C9-ALS/FTD organoids. Transcriptomics analysis identified the PU.1 (*SPI1*) regulon as the most strongly deregulated TF network in C9-oMGs and viral-mediated PU.1 expression rescued phagocytotic and gene expression deficits. Overall, our results show that in a complex 3D disease environment composed of different cell types, C9-HRE leads to reduced microglial functions. Further, our data identify PU.1 as a potential target for restoring microglial dysfunction in C9-ALS/FTD.

An increasing number of studies implicate microglial dysfunction in the pathogenesis of C9-ALS/FTD.^5^ Both reduced and increased microglial activity have been reported, likely dependent on differences in experimental settings, species, and pathogenic mechanisms and disease stage studied. Because part of this previous work indicated that the diseased microenvironment may contribute to the dysregulation of microglia in C9-ALS/FTD, we exploited a neural organoid model in which microglia develop innately and in which C9-HRE pathology is present.^22^ Furthermore, we confirmed two frequently studied C9-HRE pathologies in microglia in oMGs,^4,27,58^ i.e. RNA foci and reduced C9ORF72 expression. C9ORF72 expression was reduced at the protein but not mRNA level. Although DPRs have been reported in microglia,^59-62^ future work is needed to establish their presence in oMGs. The use of organoids allowed us to carefully assess for the first time, human microglia 3D morphology at early disease stages (i.e. as compared with post-mortem analysis). In general, microglia reacting to pathological stimuli retract their branches and assume an ameboid shape, which is associated with increased phagocytosis.^28,29,37^ Our analysis showed a subtle decrease in C9-oMG ramification, in line with observations in C9-ALS post-mortem tissue studies,^6,7,63^ in addition to reduced levels of the post-synaptic protein PSD-95 in oMGs in organoids. While C9-ALS/FTD cerebral organoids showed reduced overall PSD-95 expression,^22^ normalized levels of this synaptic protein were reduced in oMGs. This together with our transcriptomics and live cell imaging data, and work by others,^15^ indicate a reduced ability for phagocytosis by C9 microglia. Similar to our observations, reduced microglial complexity was also found in monocyte-derived microglia-like cells (MDMi) from sporadic ALS patients, concomitant with decreased phagocytic capacity and altered cytokine secretion.^64^ It is therefore possible that ALS is characterized by a distinct relationship between morphology changes and function, i.e reduced complexity and phagocytosis, as compared with other diseases. In future studies, it would also be interesting to investigate whether C9 microglia show any dystrophic properties, as dystrophic microglia have been described in neurodegenerative diseases and are linked to loss of function, impaired phagocytosis and senescence.^65-68^

The microglia transcriptomic signature of C9-oMGs closely resembles the signatures recently reported in microglia in human post-mortem brain and spinal cord tissue from C9-ALS/FTD patients.^14^ In this study, C9 microglia displayed reduced gene expression related to phagocytic and lysosomal pathways, and an impaired transition into DAM and HLA states. Interestingly, this reduction was more pronounced in the spinal cord as compared with the motor cortex. As several protocols are available for generating neuromuscular or spinal cord organoids,^69,70^ it will be interesting to determine whether microglia added to these organoid models show reduced C9-ALS responses similar to those observed in our study. Comparison of C9-oMG expression profiles to a known DAM signature^43^ also showed downregulation of many DAM genes and a defective DAM transition. These changes included downregulation of *TREM2*, and other major microglia genes such as *SPI1*, *TMEM119* and *TLR4*. TREM2 deficiency in microglia has previously been linked with a loss of DAM responses^71^ and TREM2 has been proposed as an inducer of DAM and HLA states of microglia reacting to Aβ plaques.^72^

In addition to downregulated genes, a large number of genes displayed increased expression in C9-oMGs, especially those related to synaptic and axonal pathways. Microglia are known to contribute to synaptic processes such as synaptogenesis or pruning,^48^ and the observed gene expression changes may relate to the synapse loss and dysfunction reported in C9-ALS.^73,74^ It is possible that upregulation of synaptic genes in C9-oMGs is part of a compensatory mechanism to maintain synapses. C9-ALS/FTD has also been linked to altered neurogenesis and reduced cortical and thalamic size *in utero.*^75^ These developmental effects could influence microglial responses and, for example, trigger expression of developmental and axonal genes in C9-oMGs. Future studies are needed to establish the functional consequences of enhanced microglial expression of synaptic and axonal genes in C9-ALS/FTD.

Recent RNA-seq analysis of 2D iPSC-derived C9 microglia reported mild gene expression changes, without major changes in microglia-related genes.^16,17^ In contrast, our analysis of C9-oMGs identified widespread transcriptomic changes related to, for example, lysosomal, inflammatory and phagocytic pathways. This apparent discrepancy may be explained by the presence (organoids) or absence (2D cultures) of a disease microenvironment containing other cell types. This hypothesis seems to be supported by the fact that LPS priming (i.e. an external stimulus) induces larger gene expression changes in 2D iPSC-derived C9 microglia.^16^ In addition, several studies suggest that organoid-grown microglia, as compared with other microglia cultures, are more similar to microglia found *in vivo*, which may also contribute to these differences.^76,77^ A phenotype that is consistent across several C9 microglia studies is reduced phagocytosis. One possible explanation for this is that this phenotype is more dependent on intrinsic and less on extrinsic factors.

LPS treatment is used to induce microglial activation and an inflammatory response. The reported effects of various LPS treatments in different 2D iPSC-derived C9 microglia studies are diverse but range from no effects between HC and C9 microglia to very selective changes in the secretion of only a few inflammatory cues (e.g. MMP-9^16^ or IL-6 and IL-1β^15^). In line with this, LPS treatment of oMGs only elicited a differential response between HC- and C9-oMGs for 2 out of 18 secreted factors assayed (CCL2, CXCL1). However, while limited, these changes may still be significant as, for example, MMP-9 was shown to mediate LPS-induced microglia-mediated toxicity towards MNs.^16^ C9-oMG-containing organoids showed reduced secretion of most cytokines and chemokines tested under basal and LPS treated conditions. This is in line with the RNA-seq analysis revealing a downregulation of TLRs and negative enrichment for nuclear Factor kappa-light-chain-enhancer of activated B cells (NF-kβ) and mitogen-activated protein kinase (MAPK) signalling pathways. TLRs, together with PU.1, play a crucial role in the production of cytokines and chemokines.^78^ However, further work is needed to link C9-HRE to impairment of these mechanisms and dampened oMG activity. The effect of LPS treatment of organoids was modest, with only a few factors showing induced secretion (i.e. IL-6, CCL8). It is possible that limited tissue penetration of LPS contributed to this restricted response. However, gene expression analysis of C9-oMGs purified following organoid LPS treatment show an impaired LPS response for *IL-1β* and *TNF-α*, which was not detected at the secretome level. An additional explanation could therefore be that other cell types in the organoid modulate microglia cytokine release or compensate for microglial changes by differentially releasing cytokines themselves. In line with this, previous studies have shown that neurons and astrocytes influence the transcriptional state of microglia and modulate their functional activity.^79^ For example, co-culture of microglia with neurons and astrocytes, as compared with mono-cultures, impacts the inflammatory response of microglia to LPS, resulting in changes closer to the *in vivo* response.^23,79^

Several lines of evidence support reduced phagocytosis by C9-oMGs. First, RNA-seq analysis of oMGs showed a decrease in gene expression related to phagocytic pathways. Second, C9-oMGs and C9-iMGs,^15^ displayed reduced uptake of bacterial particles, an effect rescued by PU.1 re-expression. Third, LAMP1 expression and PSD-95 uptake were reduced in oMGs in C9 organoids. This phagocytic and/or lysosomal defect may hinder the clearance of toxic proteins or damaged neurons and thereby contribute to neurodegeneration in C9-ALS/FTD. Despite this defect, overall PSD-95 density in C9 organoids was significantly decreased in comparison to HC. This suggests that synaptic loss is most likely unrelated to altered microglial clearance in oMG-containing C9 organoids. Different effects of C9-HRE and C9ORF72 loss-of-function on microglial phagocytosis have been reported. Two dimensional iPSC-derived C9 microglia show increased uptake of synthetic Aβ (in four out of seven C9 lines),^17^ and increased phagocytosis of pHrodo zymosan beads,^80^ while another study found impaired phagocytosis of C9 microglia, which was restored by rapamycin treatment.^15^ In *C9orf72* knock-out mice microglia were shown to support increased Aβ clearance, synaptic loss and neuronal deficits.^13^ These inconsistencies may reflect differences between mouse and human microglia,^31^ and the fact that human C9 microglia display incomplete C9ORF72 loss in addition to other C9-HRE pathologies.^15-17^

The disruption of phagocytosis and lysosomal function in oMGs is consistent with the role of C9ORF72 in endo-lysosomal and phagocytic trafficking.^81-85^ C9ORF72 deficiency has been associated with defective phagosome to lysosome maturation in bone marrow-derived macrophages^11^ and *Caenorhabditis (C.) elegans.*^86^ Additionally, loss of C9ORF72 has been described to impair phagolysosome acidification.^87,88^ Multiple phagocytic receptor and regulatory genes, e.g. TREM2, TLR4, TLR3, MARCO, SYK, CD33, CD36, were affected in C9-oMGs. To identify more upstream gene expression changes that could govern these phagocytic pathway alterations we performed DoRothEA analysis. This identified a regulon driven by PU.1 (*SPI1*), a major regulator of myeloid development and function, as the most strongly deregulated TF network. Several previous studies have implicated loss of PU.1 in reduced phagocytosis by primary human microglia^52^ and BV-2 cells.^55,57^  *Vice versa*, increased PU.1 expression enhanced phagocytosis of zymosan particles in BV-2 cells and induced elevated cytokine production.^55^ Furthermore, subtle changes in PU.1 expression can induce large transcriptomic changes in microglia,^89^ and PU.1 knockdown in primary human mixed glial cultures triggered reduced expression of genes involved in phagocytosis and antigen presentation.^55^ In line with these observations, viral re-expression of PU.1 in microglia restored impaired phagocytosis of bacterial particles and the expression of *TREM2*, a downstream target linked to phagocytosis. Together, these data suggest that changes in the PU.1 regulon may contribute to different phenotypes observed in C9-oMGs. The ability of PU.1 re-expression to rescue phagocytic and gene expression defects in microglia, and induce cytokine production,^55^ supports the idea that PU.1 expression may be a valuable therapeutic target for treating microglial defects in C9-ALS/FTD, as suggested for Alzheimer's disease.^56^ In future studies it will therefore be interesting to explore whether, and if so how, PU.1 regulon expression is also altered in human brain tissue.

In conclusion, our data show a series of phenotypic changes in C9-oMGs that support reduced phagocytic capacity and a dampened immune response. Although neural organoids, and most likely most iPSC-derived cell types, do not represent models of fully matured brain cells, organoid tissue closely resembles the multicellular environment of the human brain and promotes (non)neuronal maturation (including microglial maturation). Further, these models facilitate important analyses, such as of microglia morphology and protein content in a 3D environment. Our data emphasize the impact of the multicellular disease environment on microglia in C9-ALS-/FTD and identify microglia-containing neural organoids as a valuable model for further studying C9 microglia and for converging different findings. Further, our results suggest that increasing the expression of PU.1, a master regulator of different microglial functions, may be a valid therapeutic approach to target microglial dysfunction in C9-ALS/FTD.

## Supplementary Material

awaf340_Supplementary_Data

## Data Availability

All data supporting the findings of this study are available within the article and its Supplementary information. The RNA-seq data discussed in this publication have been deposited in the National Center for Biotechnology Information's (NCBI) Gene Expression Omnibus and are accessible through GEO Series accession number GSE284339: (https://www.ncbi.nlm.nih.gov/geo/query/acc.cgi?acc=GSE284339).
