# Supplementary material for: PU.1 restores microglial dysfunction caused by C9ORF72 repeat expansions in neural organoids
Source: Brain. 2025 Sep 12;149(3):801–17. doi: 10.1093/brain/awaf340 (PMC13016731; doi:10.1093/brain/awaf340)
Supplement: awaf340_Supplementary_Data [file awaf340_supplementary_data.zip › brain-2025-00428-File010.pdf]

SUPPLEMENTARY INFORMATION

SUPPLEMENTARY FIGURES

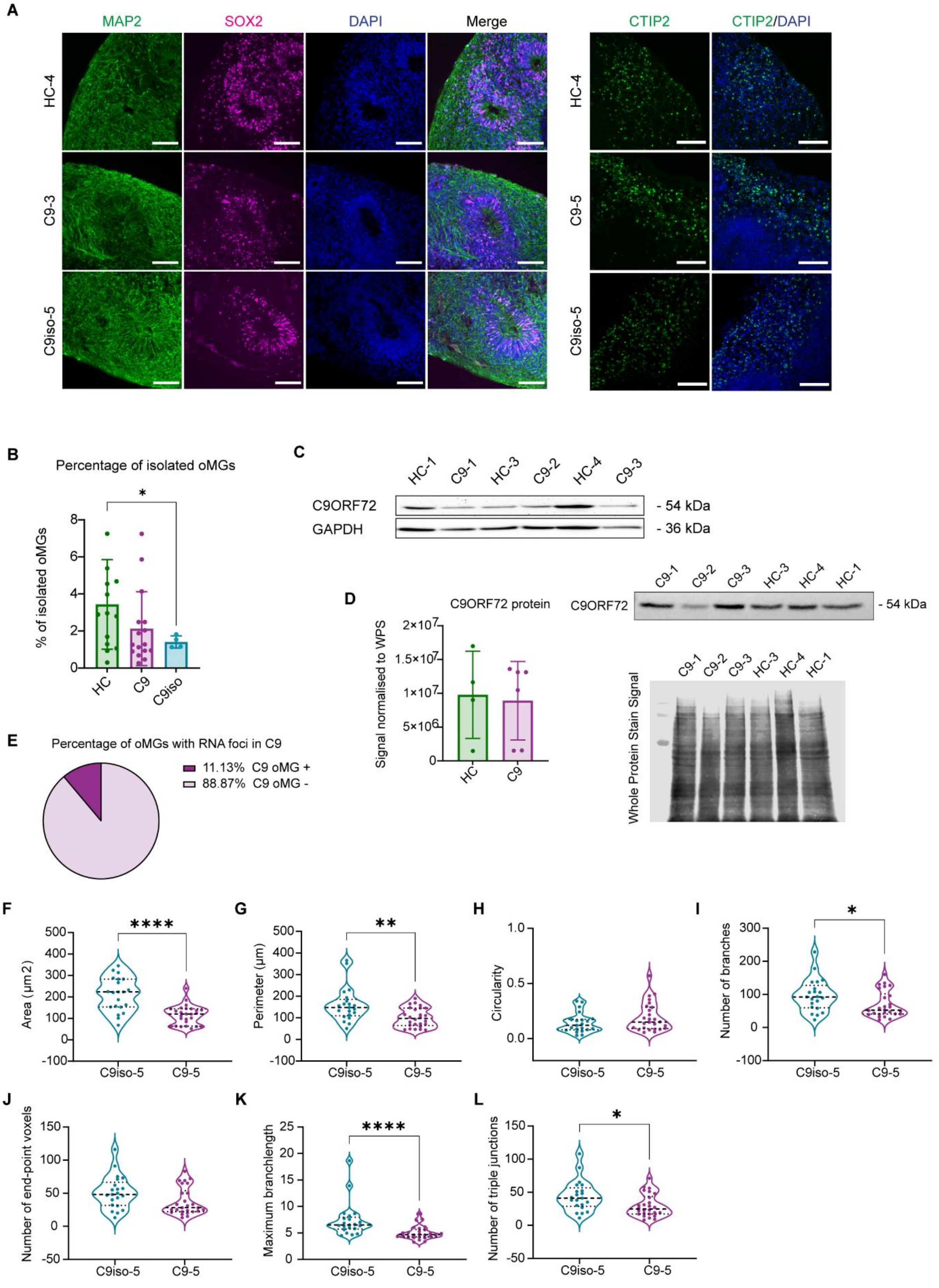

Supplementary Fig. 1: Characterization of microglia-containing organoids from healthy & isogenic control and C9ORF72-ALS/FTD iPSCs.

A Representative image showing immunohistochemistry for SOX2, MAP2 and CTIP2 on days in vitro (DIV) 64 cerebral organoids. DAPI was used to stain all cells. Scale bar, 100 µm.

B Quantification of the percentage of isolated microglia cells through MACS from organoids for HC (*n*=3 lines, 3 differentiations), C9-ALS (*n*=3 lines, 3 differentiations) and isogenic control (*n*=2 lines, 2 differentiations). Data points show line per differentiation and means ± SD. Mann-Whitney test, *$P < 0.05$.

C Western blot analysis for C9ORF72 and GAPDH on DIV 64 for all lines.

D Left part: Quantification of C9ORF72 expression normalized to whole protein staining (WPS) from flowthrough lysates after organoid-derived microglia (oMG) isolation from organoids as shown in the right panel (HC *n*=3 lines, 2 differentiations, C9 n=3 lines, 2 differentiations). Single data points showing each differentiation per line and means ± SD. Right part: Western blot for C9ORF72 and WPS on DIV64 HC or C9 organoid flowthrough lysates after oMG isolation.

E Quantification of the number of C9-oMGs positive for sense RNA foci. A total of 467 cells were analysed from 3 different C9 lines (C9-1 *n*=3 differentiations, C9-2 *n*=2 differentiations, C9-3 *n*= 2 differentiations). HC oMGs did not show RNA foci.

F-L Quantification of different morphological features from oMG skeletons from C9 (C9-5; 26 cells from *n*=1 lines, 2 differentiations) and C9iso (C9iso-5; 21 cells from *n*=1 lines, 2 differentiations) organoids. Data show cells and means ± SD. Mann-Whitney test, *$P < 0.05$, **$P < 0.01$, ***$P < 0.001$.

A

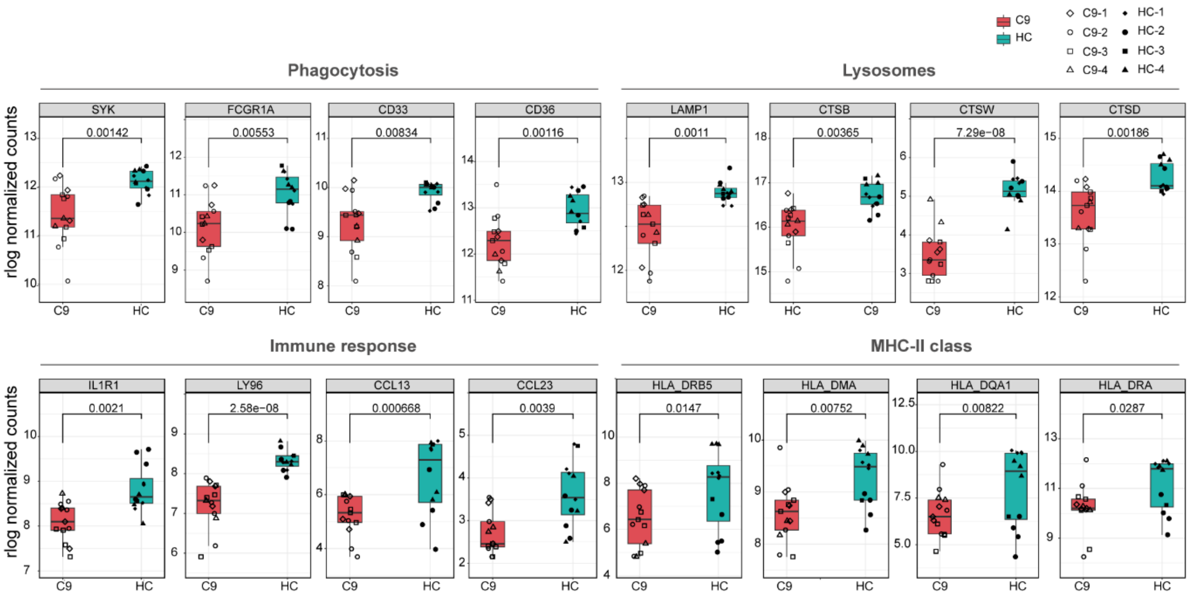

Supplementary Fig. 2: RNA-sequencing analysis of C9ORF72-ALS/FTD organoid-derived microglia reveals changes in microglia-related genes.

A Plots of rlog normalized counts showing changes in gene expression related to phagocytosis (*SYK*, *FCGR1A*, *CD33*, *CD36*), lysosome (*LAMP1*, *CTSB*, *CTSW*, *CTSD*), immune response (*IL1R1*, *LY96*, *CCL13*, *CCL23*) and MHC-II class (*HLA-DRB5*, *HLA-DMA*, *HLA-DQA1*, *HLA-DRA*) between C9 and healthy control (HC) oMGs. *P*-values are indicated.

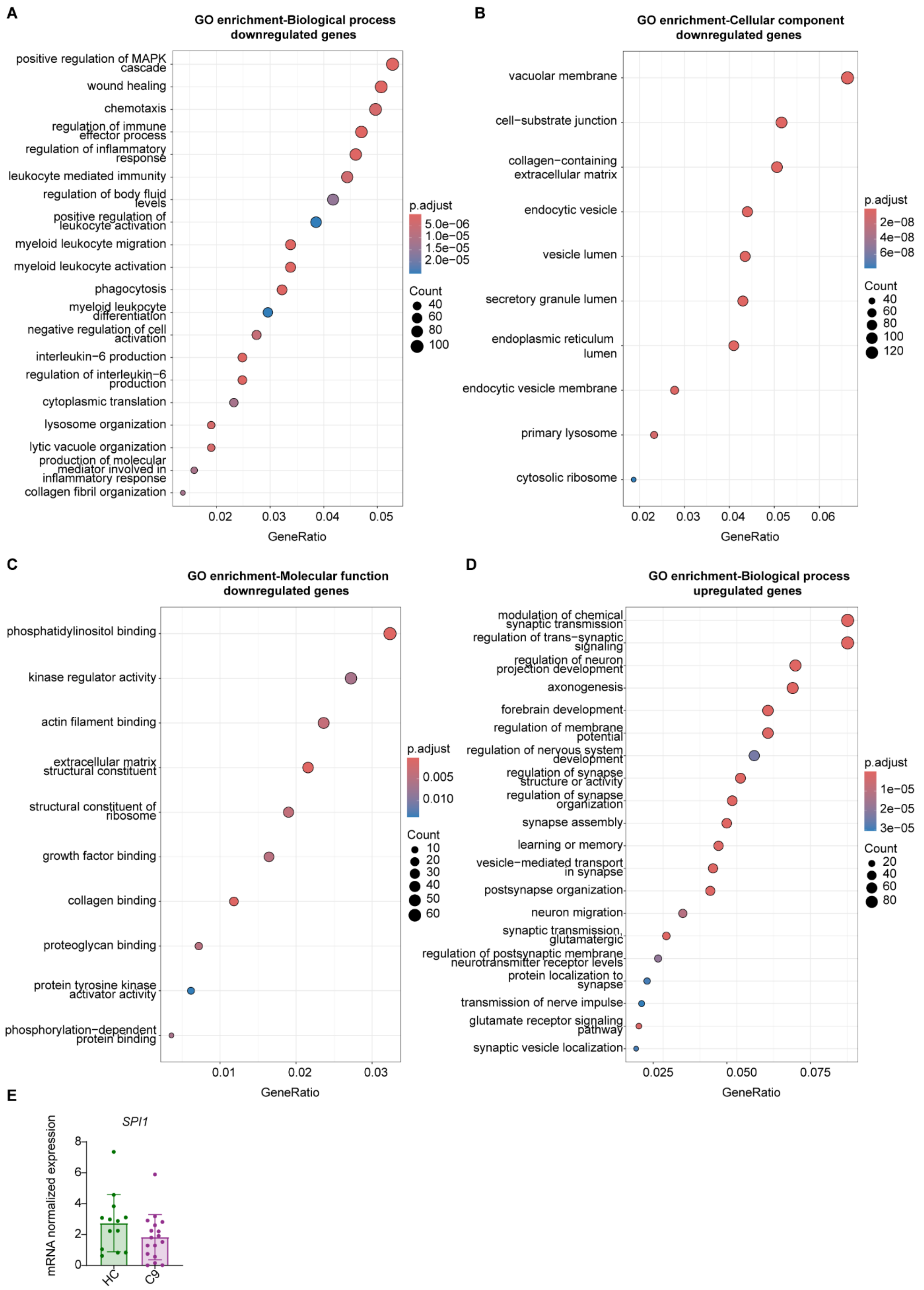
A
GO enrichment-Biological process downregulated genes
positive regulation of MAPK cascade
wound healing
chemotaxis
regulation of immune effector process
regulation of inflammatory response
leukocyte mediated immunity
regulation of body fluid levels
positive regulation of leukocyte activation
myeloid leukocyte migration
myeloid leukocyte activation
phagocytosis
myeloid leukocyte differentiation
negative regulation of cell activation
interleukin−6 production
regulation of interleukin−6 production
cytoplasmic translation
lysosome organization
lytic vacuole organization
production of molecular mediator involved in inflammatory response
collagen fibril organization
0.02 0.03 0.04 0.05
GeneRatio
p.adjust
5.0e−06
1.0e−05
1.5e−05
2.0e−05
Count
40
60
80
100
B
GO enrichment-Cellular component downregulated genes
vacuolar membrane
cell−substrate junction
collagen−containing extracellular matrix
endocytic vesicle
vesicle lumen
secretory granule lumen
endoplasmic reticulum lumen
endocytic vesicle membrane
primary lysosome
cytosolic ribosome
0.02 0.03 0.04 0.05 0.06
GeneRatio
p.adjust
2e−08
4e−08
6e−08
Count
40
60
80
100
120
C
GO enrichment-Molecular function downregulated genes
phosphatidylinositol binding
kinase regulator activity
actin filament binding
extracellular matrix structural constituent
structural constituent of ribosome
growth factor binding
collagen binding
proteoglycan binding
protein tyrosine kinase activator activity
phosphorylation−dependent protein binding
0.01 0.02 0.03
GeneRatio
p.adjust
0.005
0.010
Count
10
20
30
40
50
60
D
GO enrichment-Biological process upregulated genes
modulation of chemical synaptic transmission
regulation of trans−synaptic signaling
regulation of neuron projection development
axonogenesis
forebrain development
regulation of membrane potential
regulation of nervous system development
regulation of synapse structure or activity
regulation of synapse organization
synapse assembly
learning or memory
vesicle−mediated transport in synapse
postsynapse organization
neuron migration
synaptic transmission, glutamatergic
regulation of postsynaptic membrane neurotransmitter receptor levels
protein localization to synapse
transmission of nerve impulse
glutamate receptor signaling pathway
synaptic vesicle localization
0.025 0.050 0.075
GeneRatio
p.adjust
1e−05
2e−05
3e−05
Count
20
40
60
80
E
SPI1
mRNA normalized expression
0
2
4
6
8
HC
C9

Supplementary Fig. 3: GO enrichment analyses for differentially expressed genes in C9ORF72-ALS/FTD organoid-derived microglia.

A-C Dotplots representing top significantly enriched GO pathways for Biological Process, Cellular Component and Molecular Function after over-representation analysis (ORA) of downregulated genes for DEGs between HC and C9-oMGs using clusterProfiler package. X-axis is showing GeneRatio. Corrected *P*-values for each term are represented as colours.

D Dotplot representing top significantly enriched GO pathways for Biological Process, after over-representation analysis (ORA) of upregulated genes for DEGs between HC and C9-oMGs using clusterProfiler package. X-axis is showing GeneRatio. Corrected *P*-values for each term are represented as colours.

E RT-qPCR for *SPI1* normalized to the housekeeping gene *RPII* for HC oMGs (HC; *n*=3 lines, 3-6 differentiations) and C9-ALS/FTD oMGs (C9; *n*=4 lines, 1-7 differentiations). Single data points represent each differentiation per line and means ± SD. Mann-Whitney test, ns.

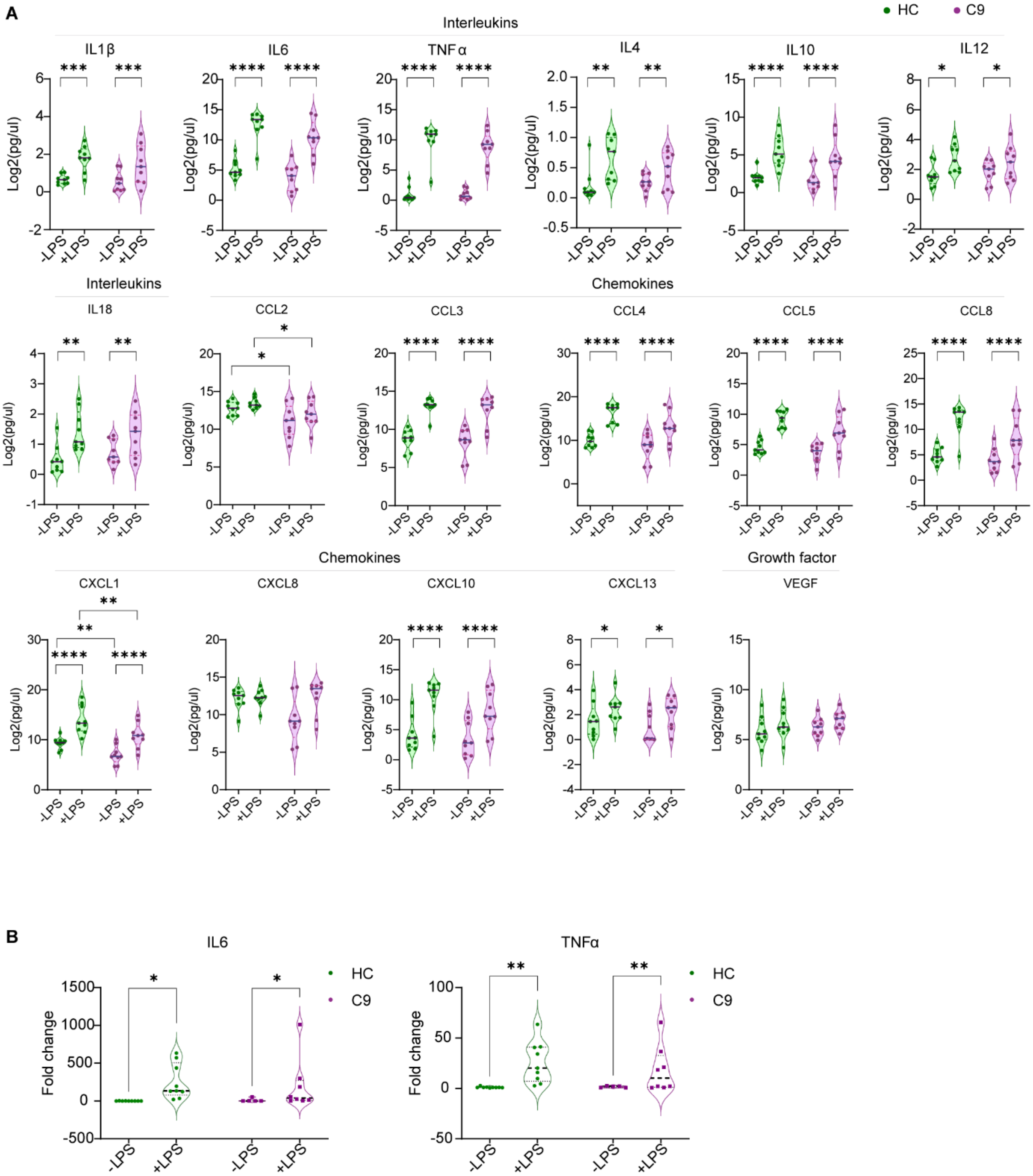
A
HC
C9
Interleukins
IL1β
IL6
TNFα
IL4
IL10
IL12
IL18
Chemokines
CCL2
CCL3
CCL4
CCL5
CCL8
CXCL1
CXCL8
CXCL10
CXCL13
Growth factor
VEGF
Log2(pg/ul)
-LPS
+LPS
B
IL6
TNFα
Fold change

Supplementary Fig. 4: Analysis of the release of cytokines and chemokines from MACS-purified control and C9ORF72-ALS/FTD organoid-derived microglia.

A Cytokine/chemokine profile analysis of media from plated isolated oMGs after a 6h stimulation with 100 ng/ml LPS between HC (n = 3 lines, 2–3 differentiations) and C9 (n = 3 lines, 3 differentiations) using the Luminex xMAP Multiplex Assay technology. Data points show cytokine/chemokine concentration as Log2 (pg/µl) of each line differentiation and means ± SD. A two-way ANOVA was performed to analyse the effect of LPS stimulation and C9-disease background on cytokine/chemokine concentration, followed by Tukey's multiple comparisons test. P-values displayed in the graphs; (*$P < 0.05$, **$P < 0.01$, and ***$P < 0.001$).

B RT-qPCR analysis of plated oMGs after LPS stimulation for key cytokines *(IL6* and *TNFα)* normalized to housekeeping gene *RPII* for HC (n = 3 lines, 2–3 differentiations) and C9-oMGs (n = 3 lines, 3 differentiations). Single data points show individual line differentiation and means ± SD. Two-way ANOVA followed by Tukey's multiple comparisons test; P-values displayed in the graphs; (*$P < 0.05$, **$P < 0.01$).

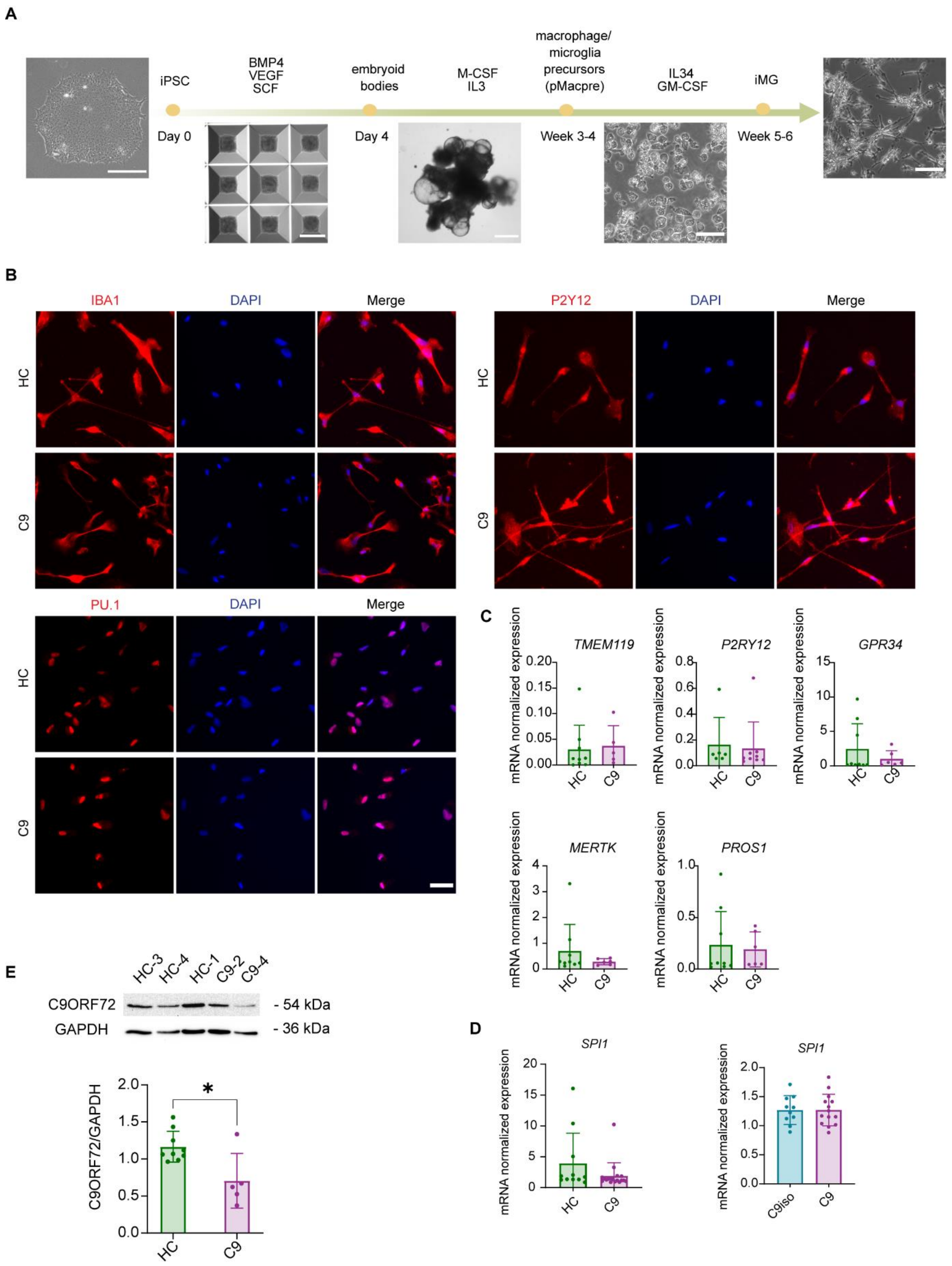
A
iPSC
Day 0
BMP4
VEGF
SCF
embryoid bodies
Day 4
M-CSF
IL3
macrophage/ microglia precursors (pMacpre)
Week 3-4
IL34
GM-CSF
iMG
Week 5-6
B
IBA1
DAPI
Merge
P2Y12
PU.1
HC
C9
C
TMEM119
P2RY12
GPR34
MERTK
PROS1
mRNA normalized expression
D
SPI1
C9iso
E
HC-3
HC-4
HC-1
C9-2
C9-4
C9ORF72
GAPDH
- 54 kDa
- 36 kDa
C9ORF72/GAPDH
*

Supplementary Fig. 5: Characterization of iPSC-derived microglia cultures.

A Illustrated timeline of direct differentiation of iPSCs into iPSC-derived microglia (iMGs) showing different stages of microglial differentiation**.** Scale bars, 500 µm for iPSC and embryoid bodies, 100 µm for pMacpre and iMGs. Image was created with Biorender.com.

B Immunofluorescent images showing expression of IBA1, P2Y12 and PU.1 in HC and C9 iMGs. DAPI was used to stain all cells. Scale bar, 30 µm.

C RT-qPCR for microglial markers *TMEM119*, *P2RY12*, *SPI1, MERTK, PROS1* and *GPR34* normalized to the housekeeping gene *RPII* for C9 (n=2 lines, 3 differentiations) and HC (n=3 lines, 3 differentiations). Single data points showing individual lines and experimental runs and means ± SD. Mann-Whitney test, ns.

D RT-qPCR for the microglial marker *SPI1* normalized to the housekeeping gene *RPII* for healthy control (HC; n=4 lines, 2-3 differentiations) and C9-ALS/FTD iMGs (C9; n=3 lines, 2-9 differentiations), left and isogenic pairs (C9iso; n=2 lines, 3-7 differentiations and C9; n=2 lines, 6-9 differentiations), right. Single data points showing individual lines and experimental runs and means ± SD. Mann-Whitney test, ns.

E Left: Western blot analysis for C9ORF72 and GAPDH on iMGs. Right: Quantification of C9ORF72 expression normalized to GAPDH as in the left panel for HC (n=3 lines, 3 differentiations) and C9 (n=2 lines, 2-3 differentiations). Single data points showing individual lines and experimental runs and means ± SD. Mann-Whitney test, *$P < 0.05$.

Created in BioRender. Pasterkamp, J. (2025) https://BioRender.com/j9bkiee

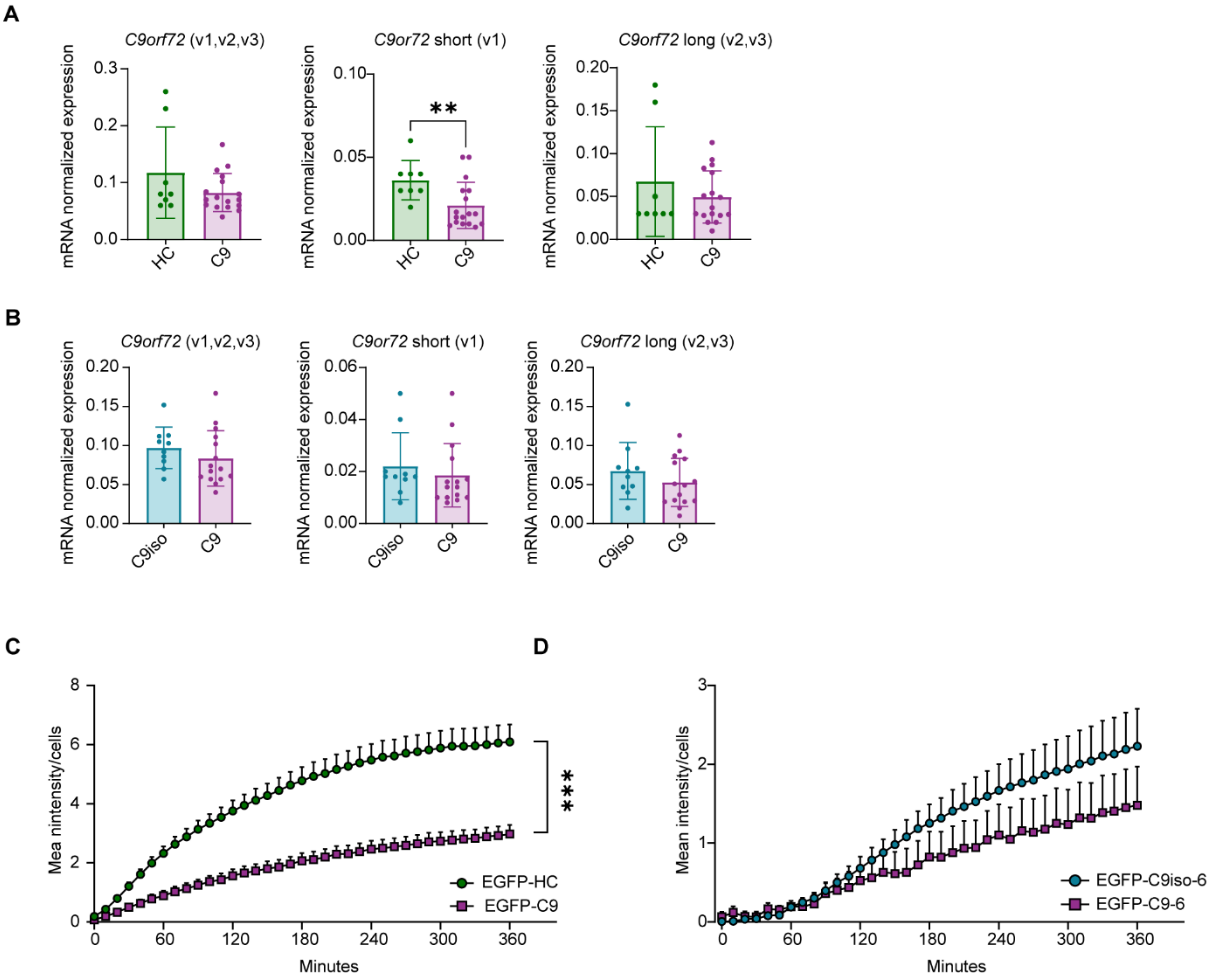

Supplementary Fig. 6: Analysis of *C9ORF72* mRA expression and phagocytosis in iMGs. **A** RT-qPCR for *C9ORF72* and *C9ORF72* variants *(C9ORF72-short(v1)* and *C9ORF72-long(v2, v3))* normalized to the housekeeping gene *RPII* for healthy control iMGs (HC; *n*=4 lines, 1-3 differentiations) and C9-ALS/FTD iMGs (C9; *n*=3 lines, 2-9 differentiations). Single data points represent each differentiation per line and means ± SD. Mann-Whitney test, ns. **B** RT-qPCR for *C9ORF72* and *C9ORF72* variants *(C9ORF72-short(v1)* and *C9ORF72-long(v2, v3))* normalized to the housekeeping gene *RPII* for isogenic pairs (C9iso; n=2 lines, 3-7 differentiations and C9; n=2 lines, 6-9 differentiations). Single data points represent each differentiation per line and means ± SD. Mann-Whitney test, $**P < 0.01$.

C Quantification of live cell imaging of pHrodo uptake, measured as mean intensity per number of cells per frame in 10 min intervals for a total of 360 min for EGFP-HC (*n*=3 lines, *n*=2-3 differentiations, *n*=4-5 image frames per line, per differentiation) and EGFP-C9 (*n*=3 lines, *n*=3 differentiations, *n*=3-5 image frames per line, per differentiation). Data are represented as means ± SEM; Two-way ANOVA and Šidák multiple comparisons test, ***$P < 0.001$.

D Quantification of live cell imaging of pHrodo uptake, measured as mean intensity per number of cells per frame in 10 min intervals for a total of 360 min for the isogenic pair EGFP-C9iso-6 (*n*=2 differentiations, *n*=5 image frames per line, per differentiation) and EGFP-C9-6 (*n*=2 differentiations, *n*=5 image frames per line, per differentiation). Data are represented as means ± SEM; Two-way ANOVA and Šidák multiple comparisons test, ns.

Supplementary Table 1 - iPSC lines

Condition	Name in paper	Gender	Age at sampling	C9 repeat size		Reference
				allele 1	allele2	
HC	HC-1	Male	62	2	2	Harschnitz et al. (Annals of neurology, 2016); Ormel et al. (Nature Communications, 2018), (iPSC 1)[1,2]
HC	HC-2	Male	49	5	6	Ormel et al. (Nature Communications, 2018), (iPSC 5)[2]
HC	HC-3	Female	60	2	2	Meyer et al. (Cell Reports, 2019), (NL1)[3]
HC	HC-4	Male	64	5	12	Meyer et al. (Cell Reports, 2019), (NL2)[3]
C9-ALS	C9-1	Female	46	13	961	Shi et al. (Nature Medicine, 2018; https://www.coriell.org/0/Sections/Search/Sample_Detail.aspx?Ref=ND06769)[4]
C9-ALS	C9-2	Male	49	2	809	Shi et al. (Nature Medicine, 2018; https://www.coriell.org/0/Sections/Search/Sample_Detail.aspx?Ref=ND12099)[4]
C9-ALS	C9-3	Female	52	5	970	Shi et al. (Nature Medicine, 2018; https://www.coriell.org/0/Sections/Search/Sample_Detail.aspx?Ref=ND10689)[4]
C9-ALS	C9-4	Male	47	2	1175	https://biomanufacturing.cedars-sinai.org/product/cs29ials-c9nxx/ ; Sareen et al. (Science Translational Medicine, 2013)
C9-iso	C9iso-4	Male	47	0	0	https://biomanufacturing.cedars-sinai.org/product/cs29ials-c9n1-isoxx/
C9-ALS	C9-5	Male	57	2	886	https://biomanufacturing.cedars-sinai.org/product/cs52ials-c9nxx/ ; Sareen et al. (Science Translational Medicine, 2013)
C9-iso	C9iso-5	Male	57	0	0	https://biomanufacturing.cedars-sinai.org/product/cs52ials-c9n6-isoxx/
C9-ALS	C9-6	Female				
C9-iso	C9iso-6	Female				

Supplementary Table 2 - Cytokine/Chemokine analysis

Condition	iPSC line	IL1b	IL4	IL6	IL10	IL12	IL18	IL23	TNFa	CCL2	CCL3	CCL4	CCL5	CCL8	CXCL1	CXCL8	CXCL10	CXCL13	VEGF
HC -LPS	HC-1	1.110	0.115	6.552	0.892	2.757	1.226	5.894	1.939	11.641	7.834	8.271	4.741	4.087	7.984	10.448	7.606	2.774	10.078
	HC-1	1.308	0.108	7.461	0.879	2.897	1.027	6.261	3.672	11.661	8.832	9.197	4.689	4.184	8.554	11.286	7.672	2.124	10.027
	HC-3	0.753	0.065	7.697	0.847	1.248	0.704	7.025	4.064	11.828	9.181	10.959	5.130	5.607	7.472	10.863	7.743	2.116	10.408
	HC-3	0.566	0.050	4.508	0.020	1.186	0.633	5.254	0.217	11.150	5.589	6.778	3.438	2.313	6.409	8.472	6.000	2.568	11.345
	HC-4	1.068	0.193	5.633	0.661	1.739	1.717	4.530	1.228	11.468	5.116	7.286	3.918	1.829	8.262	10.087	7.576	2.381	2.829
	HC-4	2.255	0.189	7.360	0.715	1.887	3.042	5.179	1.443	11.922	4.680	7.534	4.798	5.104	9.731	11.776	9.148	3.068	2.526
	HC-4	1.659	0.340	7.620	1.253	2.745	1.757	6.193	2.137	12.123	6.749	8.579	5.312	5.337	9.624	13.399	9.139	2.225	3.611
HC +LPS	HC-1	0.835	0.147	8.195	1.234	2.280	1.342	5.287	2.796	11.758	8.214	11.200	5.756	6.134	8.918	13.494	8.829	3.223	9.861
	HC-1	1.469	0.079	8.788	1.710	2.041	0.927	6.729	3.993	11.887	10.000	11.139	6.171	6.040	9.222	12.494	9.056	2.746	6.740
	HC-3	0.897	0.155	5.085	0.414	3.183	0.966	7.342	2.394	10.526	7.121	9.112	5.104	4.520	6.949	9.170	6.563	2.876	10.740
	HC-3	1.020	0.213	7.074	0.597	2.867	1.228	6.768	1.638	11.902	8.082	10.432	5.127	4.985	7.676	11.078	7.514	3.779	11.740
	HC-4	2.385	0.284	10.572	2.974	2.078	1.974	5.763	4.890	12.063	8.391	10.597	7.456	7.824	10.500	13.399	10.727	2.775	2.806
	HC-4	1.790	0.290	9.859	2.042	2.135	2.116	5.941	3.137	12.070	8.112	12.493	6.927	7.331	10.488	13.399	10.254	2.926	3.030
	HC-4	2.601	0.656	10.296	2.379	2.847	2.586	6.510	4.448	12.017	8.885	11.274	7.541	6.911	10.495	13.399	9.868	2.692	3.173
C9-ALS -LPS	C9-1	0.993	0.044	2.619	0.252	0.956	0.816	4.209	0.204	9.286	2.811	6.402	3.626	0.095	5.722	6.827	7.218	4.109	11.346
	C9-1	0.813	0.093	1.037	0.108	0.956	0.699	3.078	0.028	7.950	2.514	5.605	3.377	0.095	5.510	5.946	6.267	1.622	12.290
	C9-1	0.467	0.003	0.580	0.361	1.482	0.499	4.014	0.303	8.243	0.755	4.830	3.364	0.095	5.391	4.708	5.997	2.423	11.735
	C9-2	0.256	0.030	0.267	0.315	0.947	0.406	3.212	0.025	8.126	2.116	1.161	2.679	0.086	5.128	2.392	2.049	0.237	11.421
	C9-2	0.459	0.065	0.267	0.079	0.028	0.302	3.510	0.050	8.038	2.209	4.303	2.135	0.086	5.307	4.952	2.139	0.440	10.732
	C9-2	1.071	0.025	0.267	0.020	0.678	0.510	4.354	0.108	7.902	2.116	2.270	3.260	0.306	5.152	2.829	2.908	0.774	11.737
	C9-3	0.868	0.041	1.437	0.561	1.519	0.939	4.932	0.059	10.138	3.860	7.193	4.383	0.196	6.803	7.802	8.087	4.488	12.197
	C9-3	1.593	0.273	3.815	0.780	3.524	2.483	6.559	1.878	11.075	4.738	8.397	5.563	0.477	7.815	9.639	8.267	4.762	12.737
	C9-3	0.957	0.115	2.916	0.494	0.544	1.464	5.616	0.617	10.336	5.305	7.926	5.091	0.196	7.358	8.839	7.823	3.318	12.284
C9-ALS +LPS	C9-1	0.747	0.082	5.690	0.706	1.242	0.962	4.512	0.595	10.058	4.606	7.765	4.233	1.741	7.555	9.751	8.208	3.856	11.620
	C9-1	0.257	0.028	4.528	0.361	0.031	0.780	4.014	0.224	9.159	4.186	7.544	3.826	1.272	6.315	8.400	7.099	3.345	12.768
	C9-1	0.471	0.003	2.900	0.066	0.061	0.180	4.128	0.238	8.396	3.180	5.435	2.896	0.185	5.271	7.117	6.295	2.262	11.558
	C9-2	0.520	0.079	0.267	0.060	0.302	0.451	4.289	0.164	8.114	0.699	1.971	2.310	0.086	5.175	3.211	2.069	1.057	11.222
	C9-2	0.425	0.005	3.213	0.470	0.028	0.488	4.607	0.294	9.750	3.879	6.954	3.397	1.938	6.079	7.088	6.491	2.450	11.042
	C9-2	0.502	0.025	0.267	0.020	0.159	0.199	4.289	0.230	7.628	2.328	2.235	1.431	0.086	5.137	2.884	2.508	1.032	11.670
	C9-3	1.686	0.222	7.082	2.258	0.451	1.821	6.135	2.110	11.359	6.902	10.311	6.335	4.204	8.669	11.765	9.821	5.393	12.259
	C9-3	1.121	0.303	8.305	1.490	1.243	2.283	6.273	3.564	11.557	8.691	11.439	5.725	5.231	8.688	11.369	9.804	4.238	11.906
	C9-3	1.350	0.070	6.065	1.165	0.065	1.341	5.736	1.871	10.597	6.926	9.376	4.890	2.835	7.709	10.168	8.406	3.421	12.203

Supplementary Table 3 - RT-qPCR primers

Gene	Forward sequence (5’ to 3’):	Reverse sequence (5’ to 3’):
CTIP2	GGTGCCTGCTATGACAAGG	GGCTCGGACACTTTCCTGAG
GPR34	CCTGATGTCCAGTAACATTCGC	CATGCAGGGAGTATCCTGGT
IL1β	TTTGAGTCTGCCCAGTTCCC	TCAGTTATATCCTGGCCGCC
IL6	TGCAATAACCACCCCTGACC	TGCGCAGAATGAGATGAGTTG
MERTK	CTCTGGCGTAGAGCTATCACT	AGGCTGGGTTGGTGAAAACA
NEUN	TCGTAGAGGGACGGAAAATTGA	GCCGTTGGTGTAGGGGTTC
P2Y12	TTTGTGTGTCAAGTTACCTCCG	CTGGTGGTCTTCTGGTAGCG
PROS1	TTGCACTTGTAAACCAGGTTGG	CAGGAACAGTGGTAACTTCCAG
RPII	GCACCACGTCCAATGACAT	GTGCGGCTGCTTCCATAA
SOX2	CGAGGGAAATGGGAGGGGTGC	TGCAGCTGTCATTTGCTGTGGGT
SPI1	GTGCAAAATGGAAGGGTTTCCC	TACTCGTGCGTTTGGCGTTG
TBP	CCACAGCTCTTCCACTCACA	GCGGTACAATCCCAGAACTC
TMEM119	CTTCCTGGATGGGATAGTGGAC	GCACAGACGATGAACATCAGC
TNFα	TGGAGAAGGGTGACCGACTC	TCACAGGGCAATGATCCCAA
TREM2	TCAGGAAGGTCCTGGTGGA	GGGTGGGAAGGGGATTTCTC

Supplementary Table 4 - Antibodies

Primary antibodies	dilution	vendor	Cat #	RRID:
Mouse anti-C9ORF72	1000	GeneTex	GTX632041	RRID:AB_2784546
Rat anti-CTIP2	1000	Abcam	ab18465	RRID:AB_2064130
Rabbit anti-GAPDH	1000	Abcam	ab9485	RRID:AB_307275
Rabbit anti-IBA1	500	Wako Chemicals	019-19741	RRID:AB_839504
Mouse anti-LAMP1	1000	Santa Cruz Biotechnology	SC-20011	RRID:AB_626853
Chicken anti-MAP2	1000	Neuromics	CH22103	RRID:AB_2314763
Mouse anti-PSD95	500	Thermo Fisher Scientific	MA1-046	RRID:AB_2092361
Rabbit anti-PU.1	500	Cell Signaling Technology	2258	RRID:AB_2186909
Rabbit anti-SOX2	500	Abcam	ab97959	RRID:AB_2341193
Mouse anti-TUJ1	1000	BioLegend	801201	RRID:AB_2313773
Chicken anti-GFP	1000	Abcam	ab13970	RRID:AB_300798

Secondary antibodies	dilution	vendor	catalog number	RRID:
Goat anti-rabbit HRP conjugated	5000	Jackson ImmunoResearch Labs	111-035-003	RRID: AB_2313567
Goat anti-mouse HRP conjugated	3000	Jackson ImmunoResearch Labs	115-036-020	RRID: AB_2338521
Donkey anti-chicken Alexa Fluor™ 488	1000	Thermo Fisher Scientific	A78948	RRID:AB_2921070
Donkey anti-rat Alexa Fluor™ 488	1000	Thermo Fisher Scientific	A21208	RRID:AB_2535794
Donkey anti-rabbit Alexa Fluor™ 488	1000	Thermo Fisher Scientific	A21206	RRID:AB_2535792
Donkey anti-rabbit Alexa Fluor™ 568	1000	Abcam	ab175470	RRID:AB_2783823
Donkey anti-mouse Alexa Fluor™ 568	1000	Thermo Fisher Scientific	A10037	RRID:AB_11180865
Donkey anti-rabbit Alexa Fluor™ 647	1000	Thermo Fisher Scientific	A31573	RRID:AB_2536183
Sheep anti-Digoxigenin Fab fragments, POD Conjugated	500	Roche	11207733910	RRID:AB_514500

SUPPLEMENTARY MATERIALS AND METHODS (EXTENDED)

iPSC culture

All subjects have provided written informed consent and generation of iPSC lines was approved by the Ethical Medical Committee of the University Medical Center Utrecht. Control iPSC lines were derived from donors without a psychiatric or neurologic diagnosis (Supplementary Table 1). iPSCs were cultured in feeder-free conditions on Geltrex-coated dishes (Thermofisher, A1413202) in StemFlex medium (ThermoFisher, A3349401) at 37°C with 5% CO_2[1]. Cells were passaged weekly using 0.5 mM EDTA (Thermo Fisher, 15575020) and seeded in StemFlex medium supplemented with Y-27632 (4.82 µM, Axon Medchem 1683). After 24h, Y-27632 was removed from the medium. All lines were frequently tested for mycoplasma infection (Lonza, LT07-318). Repeat-primed PCR and nanopore sequencing were performed to determine C9 repeat size. Healthy control (HC) cells had <30 HRE repeats on both alleles, while C9-ALS/FTD (C9) iPSCs had an expanded repeat on one allele with a median length between 809-1175 repeats.

Cerebral organoid culture

Cerebral organoids were generated using a modified version of a previously published protocol[5]. In short, iPSC colonies were detached and dissociated to a single cell suspension using 0.5 mM EDTA (Thermo Fisher, 15575020) followed by Accutase (Innovative Cell Technologies, Inc.; AT104). 9000 cells were seeded per well in ultra-low attachment 96 well plates (Corning; 7007) in 150 µl of HuES medium (20% KOSR (Gibco; 10828028, 3% FBS (Sigma-Aldrich; F7524), 2 mM L-Glu (Gibco; 25030024), 1x MEM-NEAA (Gibco; 11140035), 3.5 µl / 100 mM 2ME in DMEM/F-12) supplemented with 50 µM Y-27632 dihydrochloride (Axon Medchem; 1683) and 4 ng/ml bFGF (Pepro-Tech; 100-18B). After 48h,

embryoid bodies were formed and medium was changed by taking out 100 µl and adding 150 µl per well. At day 4, bFGF and Y-27632 were omitted and at day 6 neural induction medium (DMEM/F-12 (Gibco; 11320074) supplemented with 1x N2 supplement (Gibco; 17502001), 2 mM L-Glu (Gibco; 25030024), 1x MEM-NEAA (Gibco; 11140035) and 1 µl/ml heparin (Sigma-Aldrich; H3149) was given. Medium was replaced every other day. At day 13, organoids were embedded in 30 µl Matrigel droplets (Corning, 356234), transferred to 6 cm Petri dishes in cerebral organoid differentiation medium consisting of equal parts of DMEM/F-12 and Neurobasal (Gibco; 21103049) medium supplemented with 0.5x N2 supplement, 0.025% human insulin (Sigma; I9278), 2 mM L-Glu (Gibco; 25030024), 0.5x MEM-NEAA, 100 U/ml penicillin/streptomycin (p/s; Gibco; 15140122), 50 mM 2ME and 1x B27 supplement without vitamin A (Gibco, 12587010). Four days later, organoids were transferred to an orbital shaker (3.5 speed; Sigma Aldrich, Z768545) and cultured in organoid differentiation medium containing B27 with retinoic acid (RA; Gibco; 17504044). Medium was changed 3 times per week. Organoids were grown for 64-70 days.

Single cell preparation and magnetic cell sorting (MACS)

Enzyme solution consisted of papain (18.6 U/ml, Worthington, LK003176) and DNAse 1 ((337 U/ml Worthington, LK003170) in 6 ml DMEM/F12. Ten to twelve organoids were collected and washed with DPBS, followed by mechanical dissociation. Organoids were then immersed in enzyme solution and incubated for 25 min at 37°C on a shaker. Every ten minutes, the organoid cell suspension was pipeted up and down 20 times in a swirling motion to dissociate clumps. After 25 minutes of incubation, 2% FBS was added to halt the enzymatic reaction, followed by filtering of organoid cell suspension through a 100 µm cell strainer (PluriSelect, c43-50100-01). The cell suspension was centrifuged at 300g for 5 min and cells were resuspended in MACS buffer (DPBS (Gibco Life technologies, MA), 2 mM EDTA (Thermo

Fisher, 15575020) and 1% FBS). MACS was used to enrich the CD11b$^+$ fraction (Miltenyi, 130049601). In short, single cell suspensions were incubated with CD11b magnetic beads in MACS buffer for 30 min at 4°C. After incubation, cells were centrifuged at 300g for 5 min at 4°C, followed by resuspension in 5 ml MACS buffer, filtered through a 70 μm cell strainer (PluriSelect, 43-50070-01), ran over magnetic columns (Miltenyi Biotec, 130-042-401), and washed three times with MACS buffer. The CD11b$^+$ cell fraction was collected in microglia culture medium (RPMI 1640 (Life technologies, 21875034), 10% FBS, 2 mM L-Glutamine, 100 U/ml penicillin, 100 μg/ml streptomycin (BioWhittaker, Belgium) and 100 ng/ml IL-34 (Pepro Tech, 200-34)). On average, CD11b$^+$ cells constituted 3-5% of the cell suspension. Isolated CD11b$^+$ cells were collected for RNA isolation or plated on PLL-coated plates for further culture.

LPS stimulation and multiplex cytokine detection

Whole organoids and MACS-isolated oMGs were stimulated with LPS. After one day in culture, oMGs were stimulated with 100 ng/ml LPS for 6h followed by collection of media for cytokines/chemokines analysis. Cells were lysed in 500 μl Tryzol reagent for RNA isolation. For whole organoid stimulation, five organoids per line were stimulated with 100 ng/ml LPS for 24h, to allow penetrance of LPS into organoid tissue. After 24h, medium was collected. Organoids were dissociated and CD11b$^+$ cells were isolated using MACS. Isolated oMGs were lysed in 500 μl Tryzol reagent for RNA isolation. Measurement of cytokines and chemokines in media was performed using Luminex xMAP technology (MultiPlex Core Facility of the UMC Utrecht). The amount of protein detected in media from plated oMGs was normalized to the number of plated oMGs for each cytokine. The amount of protein detected in media following whole organoid stimulation was normalized to average oMG numbers isolated from organoids. Results were then Log transformed and plotted using GraphPad Prism (version 9

software) and statistical significance was assessed using Two-way ANOVA (GraphPad Prism, version 9). Details of detected cytokine amounts per line are available in Supplementary Table 2.

Generation and transduction of iPSC-derived microglia (iMGs)

iPSCs were directly differentiated into microglia (iMGs) using a previously published protocol[6]. Briefly, after dissociation into a single cell suspension following Accutase treatment, 3 x 10^6 iPSCs were seeded per well in a Aggrewell800 microwell plate (StemCell Technologies; 27865) in 2 ml SF-EB medium (Stemflex supplemented with 50 µM Y-27632 dihydrochloride, 50 ng/ml BMP-4 (PeproTech, 120-05ET), 20 ng/ml SCF (Miltenyi Biotec, 130-096-692), 50 ng/ml VEGF (PeproTech, 100-20)). The next day, embryoid bodies were formed. Two thirds of the medium was changed daily. At day 4, embryoid bodies were gently transferred to 6 well plates in pMacpre differentiation medium (X-VIVO15™ (Lonza, 02-060F) supplemented with 2 mM L-Glu (Gibco, 25030024), 100 U/m p/s; Gibco; 15140122), 50 mM 2ME, 100 ng/ml M-CSF (Miltenyi Biotec, 130-096-491) and 25 ng/ml IL-3 (Miltenyi Biotec, 130-093-909)). Medium was changed weekly by replacing two thirds of the pMacpre differentiation medium for the following 3-4 weeks. Once embryonic-like macrophage precursors (pMacpre) started to emerge in the supernatant of the cultures, non-adherent pMacpres were harvested weekly by collecting the supernatant of the EB cultures. Harvested cells were plated in PLL-coated 24 well plates at a density of 100,000 cells per well in pMGL medium (Advanced DMEM/F12 (Thermo Fisher, 12634010) supplemented with 1x N2 supplement (Gibco, 17502001), 2 mM L-Glu (Gibco, 25030024), 100 U/ml p/s (Gibco, 15140122), 50 mM 2ME, IL-34 (Pepro Tech, 200-34) and GM-CSF (Miltenyi Biotec, 130-095-372)) and further maintained for 14 days to induce microglia differentiation.

For viral transduction, microglial progenitors were seeded at a density of 200,000 cells per well and the next day cells were infected with pLV[Exp]-EGFP/Puro-EF1A>hSPI1[NM_001080547.2] (VectorBuilder, VB900006-2455hhj) or pLV[Exp]-CMV>EGFP(ns):T2A:Puro (VectorBuilder; VB221214-1988tev) at MOI 2-5. After 24h, virus-containing medium was replaced with pMGL medium. Four days after infection, puromycin selection (1 µg/ml) was started for two days and cells were maintained for up to two weeks.

Phagocytosis assay and analysis

Isolated oMGs and iMGs were used in phagocytosis assays. Isolated oMGs or iMGs were plated on PLL-coated dark clear bottom 24 or 96 well plates (ibidi, 82426; Corning, 3603) in microglia medium, supplemented with 100 ng/ml IL-34 (Pepro Tech, 200-34) at a concentration of 100,000 cells per well. Two (oMGs) or fourteen (iMGs) days after plating pH-conjugated E.coli bioparticles (Thermo Fisher, P35361) were added (0.2 mg/ml) followed by live imaging (Leica Thunder microscope) at 37 °C and 5% CO2. Four images were taken per condition every 10 min for a total of 220 min or 360 min using a 10x objective. Experiments were replicated three times for all lines.

Images were analysed using Fiji. To determine phagocytosis flux over time, fluorescence intensity changes over time were measured per image using the following script: Image > Stacks > Plot Z-axis Profile. To measure background intensity, Set Measurements > Mean gray value was used. Eight randomized spots in areas without cells were assigned per image as ROI and mean intensity in those spots was measured over time using ROI Manager > Measure > Multi measure. Background intensity was subtracted from mean intensity per image over time. The number of cells per image were manually counted. Final intensity was normalized to number of cells per image. pHrodo area (μm^2) was calculated at 220 min based on the pHrodo red channel using Analyze > Set measurements > Area. Circularity was

determined at 220 min on the pHrodo red channel and using Analyze > Set measurements > Shape descriptors.

RNA extraction, cDNA synthesis and quantitative RT-qPCR

Cells and organoids were lysed in QIAzol reagent (Qiagen; 79306) and organoids were mechanically dissociated using an UltraTurrax (IKA; T10). Total RNA was isolated using the miRNeasy micro kit (Qiagen, 217004). In short, chloroform (1:6, Riedel-de Haën, 32211) was added per sample followed by 15 seconds of vigorous shaking and incubation for 2-3 minutes RT. Samples were then centrifuged for 15 min at 12,000 g at 4˚C. The upper transparent aqueous phase was transferred to a new Eppendorf tube after which 100% ethanol (1.5x the volume) was added. Samples were loaded in mRNAeasy micro kit columns (Qiagen miRNeasy micro kit, 217004) followed by centrifugation at 8,000 g for 30 seconds. Flowthrough was discarded and samples were incubated with DNAse I and RDD buffer (Qiagen RNase-free DNase set 50, 79254) for 15 minutes at RT. After incubation, samples were washed with RWT buffer supplemented with isopropanol (Qiagen miRNeasy micro kit, 217004). A second and third wash with RPE buffer (Qiagen miRNeasy micro kit, 217004) was performed. Finally, RNA was eluted in RNAse free water (Qiagen miRNeasy micro kit, 217004) and stored at -80˚C for storage and further processing. RNA concentration and purity was assessed using Nanodrop 2000 Spectrophotometer (Thermofisher Scientific, ND-2000).

cDNA synthesis was carried out using the Superscript IV kit (Invitrogen, 18090200). Briefly, 100 ng RNA, 500 µM random hex primers (Invitrogen, 48190011) and 10 mM dNTPs (Meridian Bioscience, BIO-39025) were mixed in cDNA strips. Samples were spun down and placed in a PCR machine for 5 minutes at 65˚C. 5x first strand buffer (Invitrogen, 18090050), superscript IV reverse transcriptase (Invitrogen, 18090050), RNAse inhibitor (Invitrogen, AM2694) and 0.1 M DTT (Invitrogen, 18090050) were added per sample mixture. PCR was

performed using the following program: 10 minutes at 23˚C, 10 minutes at 53˚C, and 10 minutes at 80˚C. cDNA was stored at -20˚C.

Quantitative real-time (q)PCR was performed with FastStart Universal SYBR Green Master (Roche). For each qPCR reaction a solution of 4 ng cDNA with 6 µl FastStart Universal SYBR Green Master (Sigma-Aldrich, 4913914001) and 1 µl of primer mix (0.5 mM per primer, see Supplementary Table 3 for primer sequences) was prepared. The qPCR reaction was performed in a QuantStudio 6 Flex Real-Time PCR system (Applied Biosystems) using the following program: 2 min at 50˚C, 10 min at 95˚C, 15 sec at 95˚C for 40 cycles and 1 min at 60˚C. All samples were run in duplicates. Melting curves were assessed for primer specificity and samples with aberrant melting curves were removed. Expression levels were determined using the $2^{\Delta CT}$method and *RPII* was used as a reference gene.

Tissue fixation and Immunocytochemistry

Organoids were fixed in 4% PFA at 4˚C for 30 min, washed 3 times with PBS and placed in 30% sucrose solution ON at 4˚C. Next, 3-4 organoids per sample were transferred to base molds (M475-1, Simport Scientific), embedded in O.C.T (23-730-625, Fisher Scientific), snap frozen in isopentane and stored at -80°C. 20 µm thick sections were obtained using a Leica CM1950 cryostat and mounted on SuperFrost Plus slides (Thermo Fisher Scientific). Sections were then blocked in 3% bovine serum albumin (BSA, Sigma-Aldrich, A4503), 1% Triton-X-100 (Sigma-Aldrich, 10789704001) and 10% normal goat serum (NGS) or donkey serum (NDS, Abcam, 7475) in PBS for 1h, followed by ON incubation with primary antibodies in blocking buffer at 4˚C (Supplementary Table 4). The next day, sections were washed three times with PBS for 15 min each and incubated with Alexa Fluor-conjugated secondary antibodies (1:1000) for 1h at RT. Sections were incubated DAPI for 5 min. Finally, sections were washed three times with PBS and mounted with FluorSave reagent (345789-20, VWR),

left to dry at RT and stored at 4°C. Similarly, plated iMGs were fixed in 4% PFA for 10 min and washed 3 times with PBS. Cells were blocked in 1.5% BSA, 0.5% Triton-X-100 and 5% NGS or NDS in PBS for 1h, followed by ON incubation with primary antibodies in blocking buffer at 4˚C and incubation with a Alexa Fluor-conjugated secondary antibodies (1:1000) for 1h at RT the next day.

For morphology and lysosomal analysis, multiple Z-stacks were obtained at 1 µm intervals using the 40x objective of a Zeiss LSM 880 confocal microscope. The 63x objective was used for imaging synapse phagocytosis with an interval of 0.32 µm between Z-stacks. ZEN software was used to acquire the images and convert Z-stacks to maximum intensity projections.

Western Blot analysis

To determine C9ORF72 levels in day 64 oMGs and two weeks matured iMGs were collected and lysed in RIPA buffer (50 mM Tris, pH.7.5, 150 mM NaCl, 0.5% NP-40, 0.5% NaDoc, 1% Triton in MilliQ) with cOmplete™ protease inhibitor (Roche, 11836170001). Lysates were vortexed and run through a syringe (25G) to fragment DNA. This was followed by incubation in a rotor for 20 min at 4°C and centrifugation at 13,200 RPM for 20 min at 4°C. The supernatant was collected and stored at -80°C. Protein samples were then diluted in a loading buffer NuPAGE LDS sample buffer (Invitrogen, NP0007) containing 10% 2-mercaptoethanol. Equal amounts of protein samples were separated in SDS-PAGE gel (10%) and transferred onto a Protran® 0.45 mm nitrocellulose blotting membranes (Cytiva™, 10600002). After blocking for 1h (in 5% milk powder, 0.01% Tween 20 in Tris-buffered saline (TBS)) at RT, membranes were incubated with mouse α-C9ORF72 primary antibody and rabbit α-GAPDH primary antibody ON at 4°C (Supplementary Table 4). The next day membranes were washed using 0.05% TBS-Tween 20 (TBS-T). Blots were stained with peroxidase-conjugated secondary

antibodies for 1 h at RT and signal was detected by incubating blots with Pierce ECL substrate (Thermo Fisher Scientific). Images were acquired using a FluorChem imaging system (Protein Simple). Individual band intensities for C9ORF72 for each sample were measured and normalized to corresponding GAPDH levels using ImageJ. Organoid flowthrough samples were normalized to WPS. Whole protein stain was done with Revert™ 700 Total Protein Stain Kit (LI-COR Bioscience; #926-11010). In short, blots were incubated for five minutes at room temperature with Revert™ 700 protein stain and washed two times for 30 seconds with wash solution. The blot was placed in MilliQ water and imaged with Odyssey® CLx imaging system (LI-COR Bioscience) at 700 nm. Subsequently, the blot was destained for five minutes at room temperature with destaining solution and washed with MilliQ water before blocking. Statistical significance of the relative expression between conditions of each protein was estimated using a T-test (GraphPad Prism version 9 software).

Fluorescent *in situ* hybridization

Locked nucleic acid (LNA) fluorescence *in situ* histochemistry (FISH) was performed as described [7]. Briefly, fresh-frozen or PFA-fixed organoids were cut into 20 µm thick sections on a Leica Cryostat. Slides were stored at -80°C until use. After pre-fixation (4% PFA for 10 min at RT), sections were acetylated (10 min at RT) and permeabilized with proteinase K (5 µg/ml for 5 min at RT). Prehybridization with hybridisation buffer for 1 h at RT was followed by the hybridization with 40 nM of a custom-made 3’ and 5’ DIG-labelled probe targeting the sense C9ORF72-HRE (Sequence: CCCGGCCCCGGCCCC, Qiagen) or a scrambled control (Qiagen) ON at 45°C. Before hybridization, probes were denatured for 30 min at 65°C in hybridization buffer and quickly placed on ice. The next day, slides were washed once with 5x SSC for 5 min and incubated in 0.2x SSC for 1.5 h at 50°C, followed by eight washes for 10 min each in B1 solution (0.1 M Tris, pH 7.5, 0.15 M NaCl) supplemented with Tween

(0.0005%). For immunohistochemistry and ISH, slides were blocked in 10% FBS in B1 buffer with Tween (0.0005%) for 1 h at RT and subsequently incubated with anti-DIG-POD (1:500; Roche Diagnostics; 11207733910) and rabbit anti-IBA1 (1:1000; Wacko Chemicals, 019-19741) antibodies in 1% BSA, 0.3% Triton-X-100 in 1x PBS ON at 4°C. The next day, tissue slides were washed three times with B1 solution (5 min each), followed by incubation with TSA™ Cyanine 3 reagent (1:50 in amplification diluent; AKOYA Biosciences; SAT704A001EA) for 10 min at RT. Then four washes in B1 buffer supplemented with 20% Tween (5 min each) were followed by incubation with secondary antibody donkey-anti-rabbit-Alexa Fluor™ 488 (1:750; Invitrogen) in 1x PBS for 1 h at RT. Finally, slides were washed twice (5 min each) with 1x PBS, incubated with 1x DAPI for 10 min at RT, and washed once for 5 min with 1x PBS. Slides were mounted with FluorSave™ reagent (Millipore) and images were acquired on a confocal microscope (Zeiss) with image acquisition software (Zen 3.3, Zeiss). oMG cells positive and negative for RNA foci were counted in C9 samples.

RNA sequencing

Sample preparation and RNA sequencing

Sample quality control, library preparation and sequencing were performed by GenomeScan BV. Sample quality control performed before library preparation and all samples showed RQN values > 9. The sample preparation was performed according to the protocol "NEBNext Ultra II Directional RNA Library Prep Kit for Illumina" (NEB #E7760S/L). Briefly, mRNA was isolated from total RNA using the oligo-dT magnetic beads. After fragmentation of the mRNA, a cDNA synthesis was performed. This was used for ligation with the sequencing adapters and PCR amplicification of the resulting product. The quality and yield after sample preparation was measured with the Fragment Analyzer. The size of the resulting products was consistent with the expected size distribution (a broad peak between 300-500 bp). Samples were

sequenced using the Illumina NovaSeq 6000, following a data quality metrics check for possible sample and barcode contamination, using third-party (FastQC v0.11.9) and in-house (FastQA v3.1.25) quality control tools. Raw sequence reads were trimmed of low-quality bases and adapter sequences with cutadapt v2.10. Presumed adapter sequences were removed from the read when the bases matched a sequence in the adapter sequence set (TruSeq adapters). Trimmed reads were mapped the human GRCh38.p13 using a short read aligner based on Burrows—Wheeler Transform (STAR2 v2.5.4) with default settings. The mapped counts for each gene were determined and summarised in a count table using HTSeq v0.11.0, which served as input for downstream RNA-Seq differential expression analysis.

Differential gene expression analysis

DESeq2 (version 1.40.2) was used for gene expression analysis in R (version 4.3.2). Counts were normalized using rlog or vst data transformation. Distance between samples was visualized using plotPCA on rlog transformed counts. Gene expression levels were corrected for batch effects. Using the Benjamini-Hochberg false discovery rate (FDR) adjusted *p* values with a cut-off $P<0.05$ or $P<0.01$ and a Log2FC>1 and Log2FC<1 were considered differentially expressed (DE). Visualisation of data was done using the ggplot2 library (version 3.4.4). Volcano plots visualizing the Log_2FC and $-Log_{10}P$ for each gene was generated using a Bioconductor package, EnhancedVolcano (version 1.18.0). Heatmap plots of matrices of selected genes were visualized using the CRAN package pheatmap (version 1.0.12).

Pathway enrichment analysis and regulon analysis

Pathway enrichment analysis was performed using clusterProfiler (version 4.7.1) in R using DEGs with $P<0.01$ and $P<0.05$. Upregulated and downregulated DEG datasets were analysed separately and over representation analysis (ORA) using both a Gene ontology (GO) annotation

and the Kyoto Encyclopedia of Genes and Genomes (KEGG) pathway database was applied. Pathway enrichment was visualized using dotplot from ggplot2 package and cnetplot to depict linkage between biological terms and genes. Assessment of transcription factor-target gene interaction was carried out using DoRothEA (version 1.12.0) in R. Regulons from the confidence levels A and B were assigned.

Morphology image analysis

To analyse microglia morphology sections were stained for IBA1 (Wako Chemicals; 019-19741) and DAPI. Microglia morphology was assessed using a macro in Fiji software 1.53q. The macro was used on the maximum intensity projection of the IBA1 channel and comprised of the following steps: convert to greys; adjust brightness/contrast (min 0 and max 50 or 100); Filter > Unsharp mask (radius 3.0, mask weight 0.6); Noise > Despeckle; adjust threshold (35,255 dark background); Binary > Close. In set measurements tool, area, shape descriptors and perimeter were selected. Next, Analyse particles tool (size (micron2) 50-infinity; circularity 0.00-1.00; show mask; display results; exclude on edges; include holes) was used to measure cell perimeters. In addition, the masks were further used to provide end-point and branch information. The masks were skeletonized (binary > skeletonize) which were then analysed (analyse > skeleton > analyse skeleton (show detailed info)).

Synapse engulfment image analysis

To assess PSD-95 content sections were stained for MAP2 (a dendritic marker), PSD95 (a postsynaptic marker), IBA1 (a microglial marker) and DAPI (Supplementary Table 4). Images were taken only in MAP2-rich areas of organoids, where microglia were also present and images were analysed using Imaris 9.8.0 software. Z-stack images taken with a 63x objective with an interval of 0.32 μm between the Z-stacks were converted to .ims files using Imaris

converter and loaded in Imaris. First, images were processed using following script: Image Processing > Gaussian Filter > Apply (for IBA1 and PSD95 channels). Every microglia cell was analysed separately. Images were cropped in distance of 15 μm around each microglia cell, to include its scanning area. Using the Surface tool, three masks were made: 1) based on the IBA1 channel, 2) based on PSD95 staining inside the IBA1 mask, by setting voxels of PSD95 outside of IBA1 surface to zero, and 3) based on PSD95 staining outside of IBA1 mask, by setting voxels of PSD95 inside of IBA1 to zero (smooth surface > surface detail 0.141 > absolute intensity > threshold 0.8-1.5). Volume was used as an output measure for PSD95 engulfed in IBA1 and the spots tool was used to measure number of PSD95 spots in surrounding microglia. Finally, internalized PSD95 volume was normalized to PSD95 spots outside of the microglia cell.

Lysosome image analysis

To evaluate the amount of lysosomal content in oMGs, sections were stained for LAMP1, IBA1 and DAPI (Supplementary Table 4). Fiji software was used for LAMP1 analysis. The maximum intensity projection of the IBA1 channel was used to set a region of interest (ROI). This was done by manually encircling the microglia and adding this to the ROI manager (Analyze→Tools→ROI manager). The IBA1 ROI was then used on the maximum intensity projection of the LAMP1 channel, to select the LAMP1 staining of a single microglia cell and the background staining was removed (Edit→Clear outside). Background staining within the ROI was removed with the thresholding tool (automated setting). A mask was created using the analyse particles tool with the following settings: size (micron2) 0-infinity; circularity 0.00-1.00; show masks; display results. Area was selected in the set measurements tool for quantification.

Statistics

Comparisons were performed according to the nature of data. In RNA and WB experiments, samples were generated by combining several individual organoids into one sample (at least 3 organoids per differentiation), while for image analysis separate cells or image frames could be analyzed. Statistical comparisons were performed with one-way ANOVA (Brown–Forsythe and Welch test), two-way ANOVA and Mann-Whitney tests (non-paired data) using GraphPad Prism software version 9 (Graphpad Software, CA), as normality assumptions were not met. Data are presented as single data points and means ± SD. Differences were considered significant when $P < 0.05$ (*$P < 0.05$; **$P < 0.01$; ***$P < 0.001$; ****$P < 0.0001$). Immunofluorescent images were analysed and quantified by using FIJI version 1.53c software (NIH, Bethesda, MD) and Imaris software (version 9.8.0). Data were plotted using GraphPad Prism software version 9. Assembly of figures was performed using Adobe Illustrator.

SUPPLEMENTARY REFERENCES

1. Harschnitz O, van den Berg LH, Johansen LE, et al. Autoantibody pathogenicity in a multifocal motor neuropathy induced pluripotent stem cell–derived model. *Ann Neurol*. 2016;80(1):71-88. doi:10.1002/ana.24680

2. Ormel PR, Vieira de Sá R, van Bodegraven EJ, et al. Microglia innately develop within cerebral organoids. *Nat Commun*. 2018;9(1):4167. doi:10.1038/s41467-018-06684-2

3. Meyer K, Feldman HM, Lu T, et al. REST and Neural Gene Network Dysregulation in iPSC Models of Alzheimer's Disease. *Cell Rep*. 2019;26(5):1112-1127.e9. doi:10.1016/j.celrep.2019.01.023

4. Shi Y, Lin S, Staats KA, et al. Haploinsufficiency leads to neurodegeneration in C9ORF72 ALS/FTD human induced motor neurons. *Nat Med*. 2018;24(3):313-325. doi:10.1038/nm.4490

5. van der Geest AT, Jakobs CE, Ljubikj T, et al. Molecular pathology, developmental changes and synaptic dysfunction in (pre-) symptomatic human C9ORF72-ALS/FTD cerebral organoids. *Acta Neuropathol Commun*. 2024;12(1):152. doi:10.1186/s40478-024-01857-1

6. Haenseler W, Sansom SN, Buchrieser J, et al. A Highly Efficient Human Pluripotent Stem Cell Microglia Model Displays a Neuronal-Co-culture-Specific Expression Profile and Inflammatory Response. *Stem Cell Reports*. 2017;8(6):1727-1742. doi:10.1016/j.stemcr.2017.05.017

7. Kan AA, van Erp S, Derijck AAHA, et al. Genome-wide microRNA profiling of human temporal lobe epilepsy identifies modulators of the immune response. *Cell Mol Life Sci*. 2012;69(18):3127-3145. doi:10.1007/S00018-012-0992-7
