# Supplementary figures and images for: PU.1 restores microglial dysfunction caused by C9ORF72 repeat expansions in neural organoids
Source: Brain. 2025 Sep 12;149(3):801–17. doi: 10.1093/brain/awaf340 (PMC13016731; doi:10.1093/brain/awaf340)

Fig 1D; Supplementary Fig. 1C

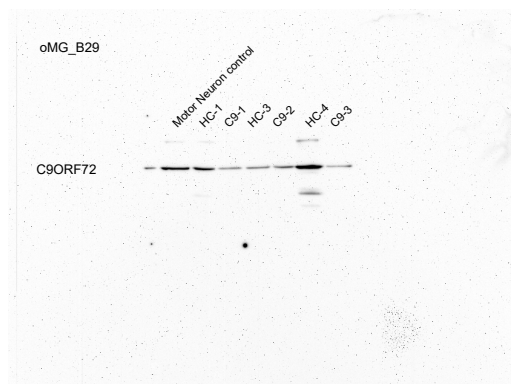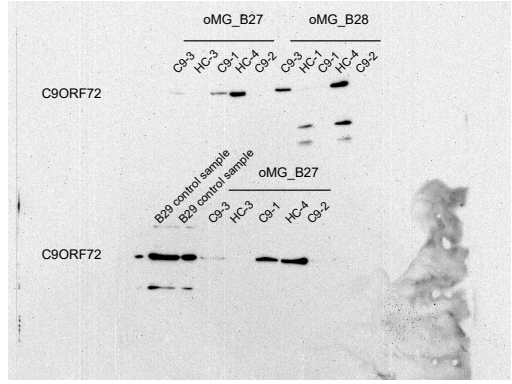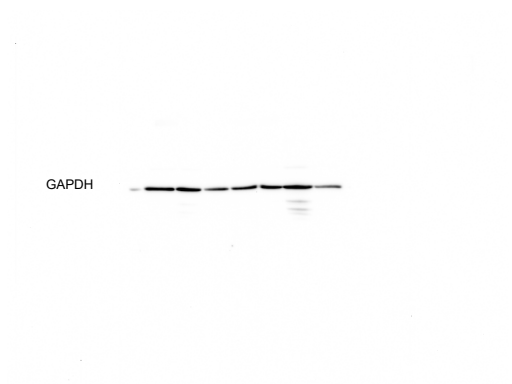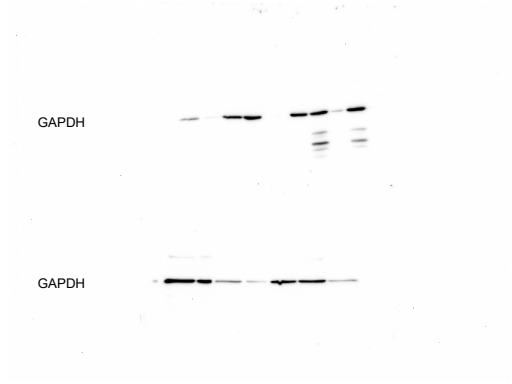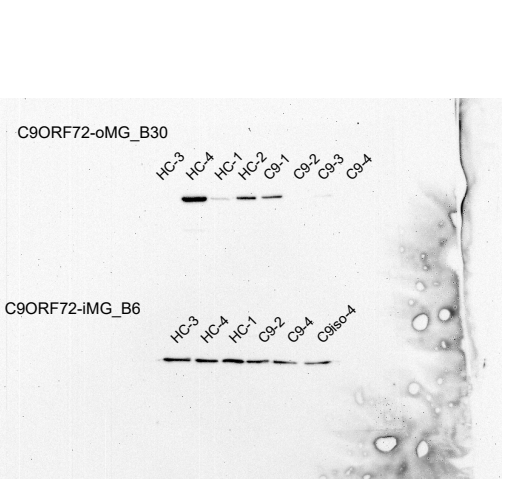

Supplementary Fig. 5E

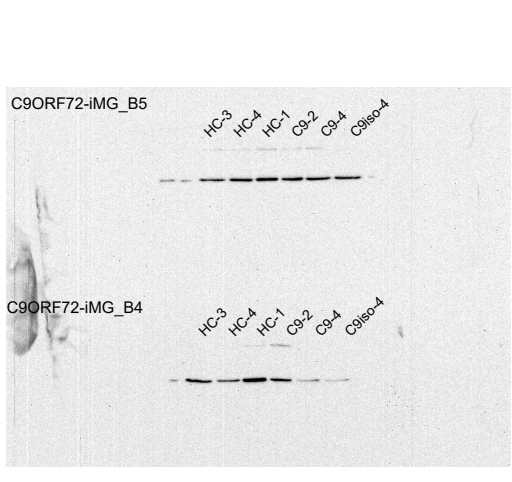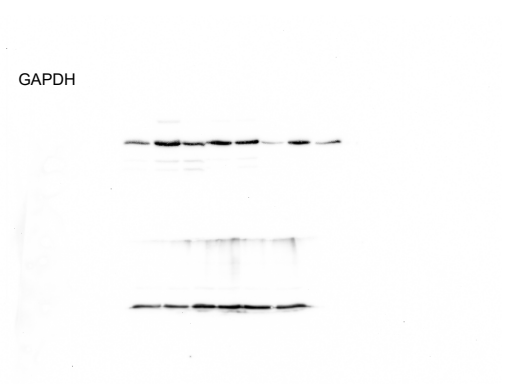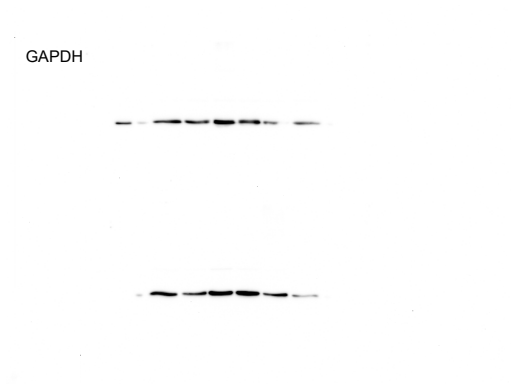

Supplementary Fig. 1D

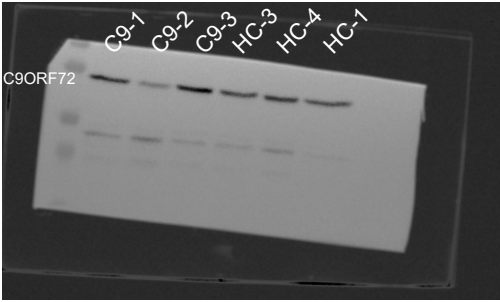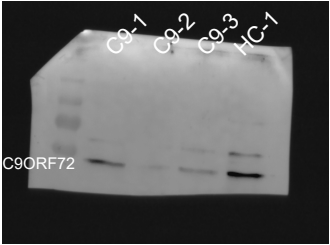

WPS

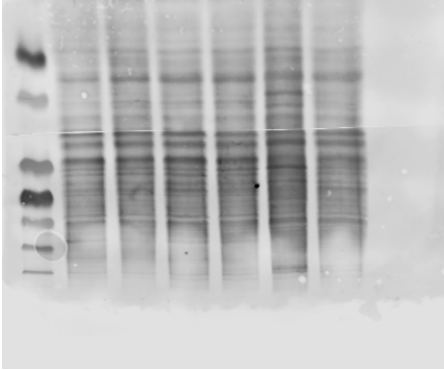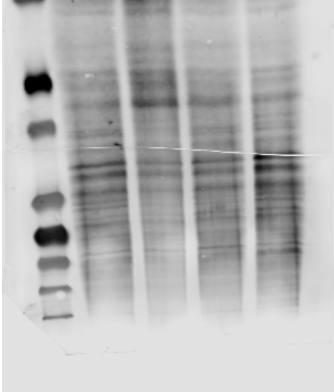

Supplement: awaf340_Supplementary_Data [file awaf340_supplementary_data.zip › brain-2025-00428-File012.pdf]
